# Structural and Functional Integration of the PLCγ Interaction Domains Critical for Regulatory Mechanisms and Signaling Deregulation

**DOI:** 10.1016/j.str.2012.09.005

**Published:** 2012-12-05

**Authors:** Tom D. Bunney, Diego Esposito, Corine Mas-Droux, Ekatarina Lamber, Rhona W. Baxendale, Marta Martins, Ambrose Cole, Dmitri Svergun, Paul C. Driscoll, Matilda Katan

**Affiliations:** 1 Institute of Structural and Molecular Biology, Division of Biosciences, University College London, Gower Street, London WC1E 6BT, UK; 2 Division of Molecular Structure, MRC-National Institute for Medical Research, Mill Hill, London NW7 1AA, UK; 3 Research Institute of Molecular Pathology, Dr. Bohr-Gasse 7, 1030 Vienna, Austria; 4 ISMB, Birkbeck College, London WC1 7HX, UK; 5 European Molecular Biology Laboratory, Hamburg Outstation c/o DESY, Notkestrasse 85, 22603 Hamburg, Germany

## Abstract

Multidomain proteins incorporating interaction domains are central to regulation of cellular processes. The elucidation of structural organization and mechanistic insights into many of these proteins, however, remain challenging due to their inherent flexibility. Here, we describe the organization and function of four interaction domains in PLCγ1 using a combination of structural biology and biochemical approaches. Intramolecular interactions within the regulatory region center on the cSH2 domain, the only domain that also interacts with the PLC-core. In the context of fibroblast growth-factor receptor signaling, the coordinated involvement of nSH2 and cSH2 domains mediates efficient phosphorylation of PLCγ1 resulting in the interruption of an autoinhibitory interface by direct competition and, independently, dissociation of PLCγ1 from the receptor. Further structural insights into the autoinhibitory surfaces provide a framework to interpret gain-of-function mutations in PLCγ isoforms linked to immune disorders and illustrate a distinct mechanism for regulation of PLC activity by common interaction domains.

## Graphical abstract

**Graphical Abstract:**

**Figure:**
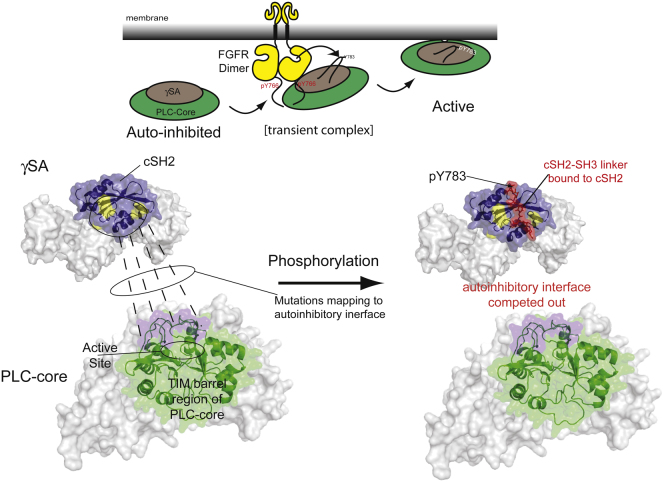

**Highlights:**

► Domains from PLCγ regulatory region show structural and functional integration ► Only cSH2 domain interacts with the PLC-core forming a high affinity surface ► Activation involves removal of autoinhibition and dissociation from the receptor ► Disease-linked mutations map to the autoinhibitory interface

## Teaser

Structural studies of multi-domain proteins provide a considerable challenge due to the presence of flexible regions not amenable to crystallography. Bunney et al. describe a strategy that generated new insights into the structural organization and integration of individual domains comprising PLCγ.

## Introduction

Selective intermolecular interactions are crucial to cellular regulatory processes and are usually mediated by modular protein domains. There are around 100 families of interaction domains in humans, each of which can be found in many copies; these therefore represent a prevalent feature of the human proteome (Liu et al., 2006; Scott and Pawson, 2009). Modules such as the Src homology 2 (SH2), Src homology 3 (SH3), and pleckstrin homology (PH) domains represent prototypes, respectively, for their involvement in recognition of specific motifs generated by posttranslational modifications, recognition of polypeptide motifs characterized by specific composition and binding to phospholipid ligands.

Taking SH2 domains as an example, there is a notable difference in the number of structures reported for the isolated domains, free or bound to phosphotyrosine (pY)-containing polypeptide ligands, compared to larger structures of proteins encompassing or interacting with this domain (Liu et al., 2006). The importance of this more comprehensive information has been recently illustrated by the finding that the selectivity of fibroblast growth factor receptor (FGFR) is controlled by a secondary SH2 domain binding site, challenging the view that short, linear polypeptides can recapitulate the SH2 domain recognition of their native targets (Bae et al., 2009). Furthermore, several structures of nonreceptor tyrosine kinases including Src, Zap70, Fes (Filippakopoulos et al., 2009), tyrosine phosphatase SHP-2 (Hof et al., 1998), and more recently of lipid kinase PI3Kβ (Zhang et al., 2011) clearly show that SH2 domains are not mere passive docking devices. Once covalently linked to other domains, SH2 domains can acquire specific intramolecular interactions that provide a means for regulation of the entire protein. Therefore, generation of comprehensive data regarding the organization and functions of the multidomain proteins is needed to further the understanding of the role of the diverse set of protein interaction domains and their specific modular function when integrated within larger polypeptides.

Most multidomain proteins have at least some degree of segmental mobility facilitated by the flexibility of the interdomain linkers (Bernadó and Svergun, 2012). This allows conformational changes that in some instances can be brought about by concerted domain movement from one distinct arrangement to another. In other cases, highly flexible linkers could result in a great number of conformations being sampled by a protein. Methodology to obtain structural insights into the organization of flexible proteins, however, still represents a challenge. Structure determination by X-ray crystallography is often either complex or not possible; however, lower resolution methods that analyze proteins in solution such as nuclear magnetic resonance spectroscopy (NMR) or small angle X-ray scattering (SAXS) provide an alternative route to understanding structural organization of these proteins (Bernadó and Svergun, 2012).

Among families of proteins involved in signal transduction, phospholipase C (PLC) enzymes that generate second messengers from phosphatidylinositol 4,5-bisphosphate (PIP_2_) incorporate a number of different protein modules. PLCγ enzymes (PLCγ1 and PLCγ2), for example, contain eight domains, four of which are unique to this PLC family (Bunney and Katan, 2011; Suh et al., 2008). The PLCγ ‘specific array’ of domains (γSA), comprising a “split” PH (spPH) domain flanking two tandem SH2 domains and an SH3 domain, is inserted between the two halves (X and Y) of the TIM-barrel catalytic domain (Bunney and Katan, 2011). As with other PLC families, PLCγ enzymes are key components of core processes in signaling networks regulated by diverse extracellular signals. Furthermore, both PLCγ enzymes have also been implicated in aberrant cellular responses linked to disease development (Bunney and Katan, 2010; Everett et al., 2009); one recent example is a link between dominantly inherited complex immune disorders and gain-of-function mutations in PLCγ2 (Ombrello et al., 2012). However, due to lack of more comprehensive structural information, the molecular aspects of the regulatory mechanisms and their subversion by gain-of-function mutations are not well understood.

For the studies reported here, we focused on PLCγ1 in the context of growth-factor stimulation. Findings describing the structural organization and integration of individual domains reveal aspects of regulatory mechanisms and critical molecular insights relevant for the function of both PLCγ enzymes and, more generally, for other multidomain proteins.

## Results

### Activation of PLCγ1 by FGFR

**Figure 1:**
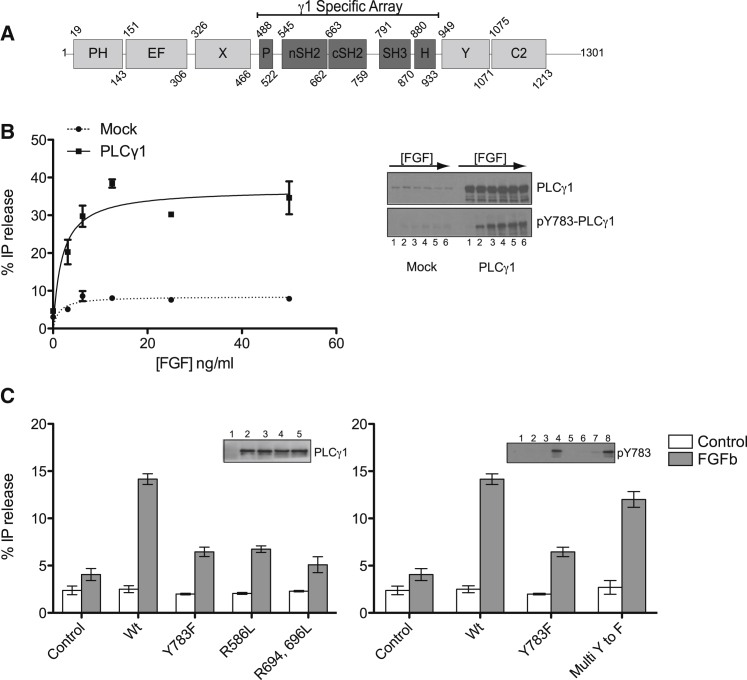
PLCγ1 Requirements for Activation by FGFR1 in PAE Cells (A) Schematic representation of the domains of PLCγ1 highlighting the γ1SA; the numbering of the amino acid residues is for human PLCγ1. (B) Dose-response curve of FGFb activation on PLCγ1. PLC activity measurements for full-length PLCγ1^wt^ were performed in PAE cells either untransfected or transfected with pTriEx4-PLCγ1^wt^ and stimulated with increasing concentrations of FGFb/Heparin. Western blotting was used to show expression of endogenous and transfected PLCγ1 as well as Y783 phosphorylation at different concentration of FGFb used for dose-response curve; lanes 1–6 correspond to 0, 3.15, 6.25, 12.50, 25.00, and 50.00 ng/ml of FGFb (inset). (C) The effects of point mutations on PLCγ1 activity were measured in PAE cells transfected with pTriEx4-PLCγ1^wt^ and constructs containing point mutations inactivating for nSH2 (R586L), cSH2 (R694L, R696L), or phosphorylation (Y783F) as well as multi Y to F (Y186, 472, 481, 771, 775, 959, 977, and 1254) replacements, with and without FGFb/Heparin stimulation. Western blotting was used to show either equal expression (lanes 1–5 correspond to control, Wt, Y783F, R586L and R694L, R696L; left inset) or phosphorylation on Y783 (lanes 1–8 correspond to eight columns in the histogram; right inset). SD is represented by error bars. See also Figure S1.

The most comprehensive studies of PLCγ enzymes have been in cellular systems that respond to growth factor stimulation and in cells from the immune system reacting to stimulation by antigens (Suh et al., 2008). Most insights into the requirements for specific domains or phosphorylation sites were obtained by introducing PLCγ variants into different cell types and inducing subsequent stimulation; this type of experiment has suggested some general similarity but also some cell system-specific features. Activation of PLCγ1 via FGFR has been clearly demonstrated and the requirement of a single phosphorylated tyrosine (pY^766^ for FGFR1) in the unstructured receptor C-terminal tail has been shown to be necessary and sufficient for the recruitment of PLCγ isoforms (Bae et al., 2009). However, the requirements for the functionality of specific regulatory modules (Figure 1A) and identification of critical phosphorylation sites for the activation of PLCγ1 have not been assessed in this system. To address this, we used a previously described porcine aortic endothelial (PAE) cell line expressing transgenic FGFR1 (Cross et al., 2000); we found that this cell line provides an excellent model where FGFR1 levels allow a physiological activation of transfected PLCγ1 by fibroblast growth factor, basic (FGFb). We measured basal as well as FGFb-stimulated PLC activity of wild-type PLCγ1 and variants with specific point mutations (Figure 1). We first established activation of PLCγ1 by FGFb in a dose-dependent manner and that this activation is consistent with increased phosphorylation of residue Y783 (Figure 1B). We then demonstrated that the functionality of both SH2 domains and phosphorylation of Y783, a residue previously implicated in activation of this enzyme by platelet-derived growth factor and epidermal growth factor (Suh et al., 2008), are also critical for full activation via FGFR1 (Figure 1C). Furthermore, based on an analysis of in vitro tyrosine-phosphorylation sites detected by mass spectrometry, we found that mutations of individual, additional phosphorylation sites (Figure S1 available online) or a combination of these sites (Figure 1C) had little effect on activation of PLCγ1. Therefore, it is unlikely that phosphorylation of any tyrosine residue other than Y783 critically contributes to activation.

The data obtained using this specific cellular signaling environment (Figure 1) provide physiologically relevant information for further structural and mechanistic studies aimed to define the roles for the implicated regulatory, modular structures and critical tyrosine phosphorylation.

### Architecture of the γSA

The key regulatory elements for activation (Figure 1) and previously described regions of autoinhibition in PLCγ enzymes (Everett et al., 2011; Gresset et al., 2010) are present within the γSA. Therefore, further mechanistic insights are crucially dependent on understanding the molecular properties of this region. Despite the information available for the isolated globular domains from the γSA (including Protein Data Bank [PDB] codes 2FJL, 3GQI, and 2HSP), the structure of this entire regulatory region has not been reported and γSA does not appear to be amenable to crystallographic approaches; this could be due to relative mobility of its modular components. To address this, we have used a combination of methods including NMR, SAXS and crystallography of regions within the γSA to obtain insights into the overall structural and dynamic characteristics of this regulatory region.

**Figure 2:**
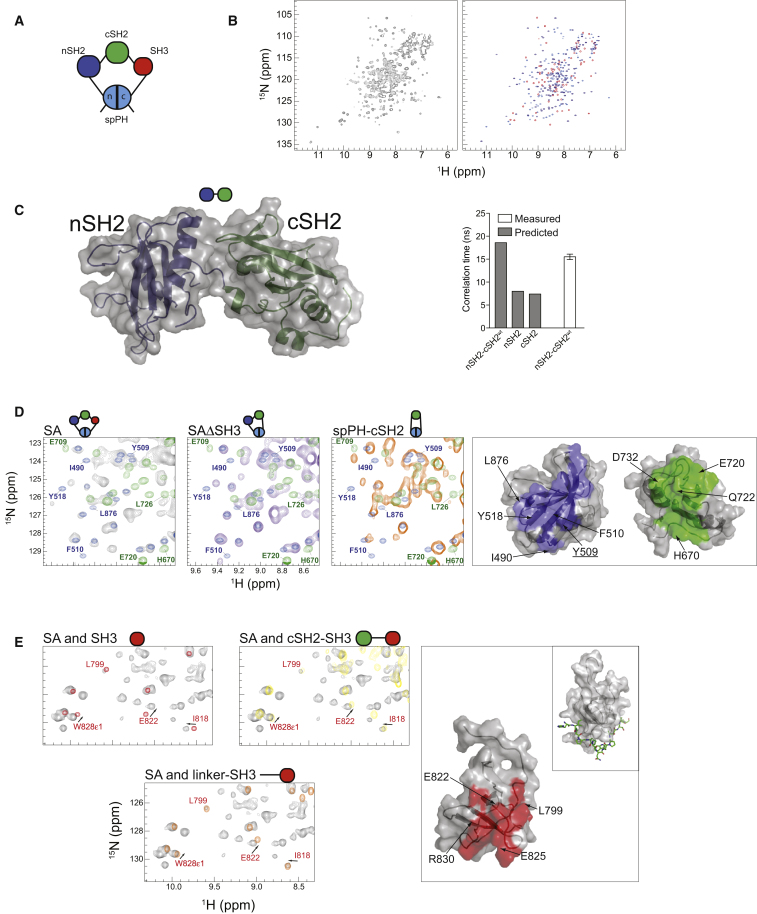
Relative Mobility and Interactions between Domains within γSA (A) Schematic representation of the domains in the γSA construct. (B) The two-dimensional ^1^H-^15^N HSQC spectra of the ^15^N-labeled γSA construct (left panel) and the overlaid spectra of the spPH, nSH2, and SH3 domains (right panel). (C) An overlaid surface and cartoon representation of the X-ray crystal structure of the nSH2-cSH2^wt^ apo-protein (left panel). A histogram showing ^15^N relaxation data recorded or predicted for the nSH2-cSH2^wt^ tandem and individual domains (right panel). (D) Overlay of regions of the ^1^H-^15^N HSQC spectra of the spPH domain (purple) and the cSH2 domain (green) with either the γSA (gray), the γSAΔSH3 (purple), or the spPH-cSH2 tandem (orange; left panels). Amino acid residues with affected chemical shifts are marked on the spectra. Surface representation showing interface residues on the spPH (blue) and cSH2 (green; right panel). Y509, mutations of which have previously been reported, is underlined. (E) Overlay of regions of the ^1^H-^15^N HSQC spectra of the γSA (gray) with the SH3 (red), cSH2-SH3 tandem (yellow) and the linker-SH3 (orange). Amino acid residues with affected chemical shifts are marked on the spectra. Surface representation of the SH3 showing amino acid residues (red) at the interface with the cSH2-SH3 linker (right panel) and the SH3 domain bound to a polyproline peptide (PDB code 1YWO; right panel inset). See also Figure S2.

The γSA construct is characterized by the pseudo-cyclic nature of the protein in which the N- and C-termini of the polypeptide are brought together in a noncovalent fashion in the context of the stable globular spPH domain (Figure 2A). In the first set of experiments shown in Figures 2 and S2, this protein and a number of derived constructs (including single, isolated domains and their combinations) were extensively characterized by 2D ^15^N,^1^H-HQSC NMR spectroscopy. In addition, we solved the structure of the tandem nSH2-cSH2 pair by X-ray crystallography (Figure 2C; Table 1).

Comparison of the NMR spectra of the γSA with those of the overlaid individual component domains (Figures 2B and S2A) reveals that the majority of the well-dispersed cross peaks are derived from the spPH, nSH2 and SH3 domains. Allowing for some chemical shift perturbations, it appears that the cross peak pattern for each of these three domains closely overlaps a subset of peaks displayed in the context of the larger protein (Figure 2B), an outcome that suggests that the overall structure of these domains is retained in the SA construct. In contrast to these three domains, the majority of the resolved cross peaks identified in the spectrum of the isolated cSH2 domain are either broad or absent from the γSA spectrum unless the γSA is bound to the SH2 domain ligand phosphopeptides (Figure S2B). There could be several reasons for these observations, however, the most likely explanation is that the line broadening is due to intermediate timescale chemical exchange resulting from transient contacts with other parts of the γSA protein that are reduced after phosphopeptide binding. Overall, the comparative analysis of the NMR spectra of isolated domains and that of γSA yielded backbone resonance assignments for over 175 residues of the larger protein (Figure S2C).

Based on the determination of the crystal structure of the PLCγ1 tandem nSH2-cSH2 domains in nonliganded form (Figure 2C, left), suggesting that these SH2 domains might possess limited intramolecular flexibility with respect to each other, we directly tested this hypothesis. We recorded ^15^N relaxation data on the tandem and isolated SH2 domains. The error-weighted mean ^15^N R_2_/R_1_ ratio (24.0 ± 0.7) obtained for 68 resolved cross peaks, is substantially larger than would be expected for the isolated domains. The corresponding derived isotropic rotational correlation time (15.5 ± 0.6 ns; TENSOR2) is closer to that predicted from the X-ray crystal structure using hydrodynamic modeling (18.6 ns; HYDRONMR) applied to residues 549–772 of the tandem than to the prediction for the isolated domains (7.4 and 8 ns) (Figure 2C, right). This suggests that the tandem construct tumbles as a more-or-less rigid entity in solution.

The comparative analysis of the NMR spectra combined with backbone NMR assignments, also revealed surfaces of individual domains likely to be involved in weak interactions within the γSA. Comparison of the γSA with deletion variants lacking either the SH3 domain (γSAΔSH3) or both the SH3 and nSH2 domains (spPH-cSH2) revealed that chemical shifts of subsets of spPH and cSH2 cross peaks are perturbed relative to the isolated domains (Figure 2D). These chemical shift perturbations correspond to nonrandom clusters of residues biased to one surface of spPH and cSH2 suggesting transient interactions between these domains both in the deletion mutants and the γSA construct. As discussed below, it has been suggested that the spPH/cSH2 interface is important in maintaining enzymatic autoinhibition (DeBell et al., 2007). Furthermore, the intramolecular surface on spPH is on the opposite side to the interaction site with Rac—determined for the spPH domain from PLCγ2 (Bunney et al., 2009)—while the interaction surface on cSH2 does not overlap with the phosphopeptide-binding pocket. Similarly, analysis of another set of constructs (Figure 2E) suggests that a cluster of residues on the surface of the SH3 domain transiently interacts with the cSH2-SH3 linker. In this case, however, the implicated surface overlaps in part with the region involved in binding of polyproline peptides (Figure 2E, inset) suggesting that proline residues from the linker might be involved in the interaction.

**Figure 3:**
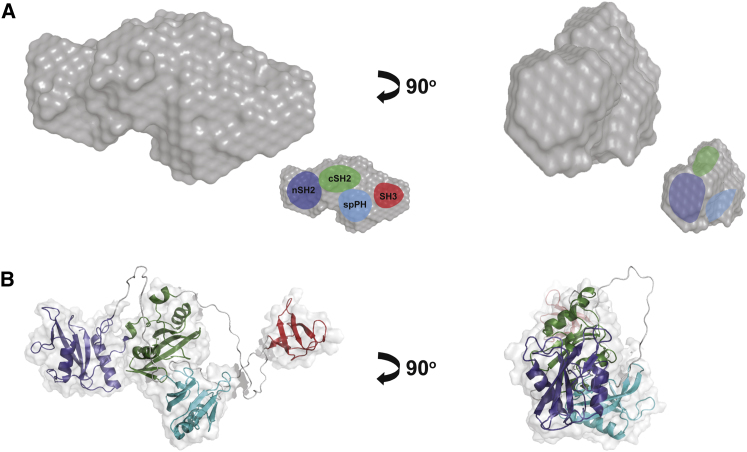
Low-Resolution “Envelope” and Overall Architecture of γSA (A) SAXS-derived envelope of the γSA. Two views of the averaged envelope (surface representation) are displayed and in the inset, the MONSA derived domain arrangement is superimposed. The envelope has a volume corresponding to the average volume of individual models. (B) Representation of an internal domain structure of the individual γSA domains, using a rigid body docking protocol implemented in the program XPLOR-NIH. Individual, structurally defined domains are shown as ribbon representations within translucent surfaces corresponding to their surface topology. The scale of this model is the same as SAXS-derived envelope shown in A. See also Figures S3 and S4 and Table S1.

In order to model γSA, SAXS data were recorded for the γSA polypeptide and a variety of related constructs (Table S1). The radius of gyration Rg and the maximum dimension Dmax values (3.2 and 10.5 nm, respectively) obtained for γSA suggest an elongated overall shape. This finding is supported by the low-resolution shapes computed ab initio from the SAXS data with DAMMIN (Volkov and Svergun, 2003); the surface of the average of several DAMMIN γSA reconstructions is shown in Figure 3A.

Given the potential for dynamic disorder suggested by NMR (Figure 2), to assess the flexibility of the protein from the SAXS data we applied the ensemble optimization method (EOM; Bernadó et al., 2007; Figure S4). The EOM analysis was performed under a series of different assumptions concerning the degree of interdomain linker dynamics. The quantitative outputs of this procedure were sensitive to the assignment of ordered and disordered regions of the linker segments. Importantly, the reconstructed distributions of the overall parameters (Rg and Dmax) are compatible with the incorporation of the contact between cSH2 and spPH domains (supported by both NMR data and SAXS data for the cSH2-spPH construct; Figure 2D; Table S1). Moreover, the Rg/Dmax distributions of the selected ensembles were narrower compared to the initial random pools (which assumed complete linker disorder) suggesting limited linker flexibility with the globular domains each sampling a relatively restricted volume (Figure S4B).

To further probe the domain organization of γSA we performed analysis of SAXS data obtained for γSA itself, γSAΔSH3 and constructs containing domain pairs nSH2-cSH2, cSH2-SH3 and spPH-cSH2. We applied MONSA (Svergun, 1999), a program that simultaneously fits multiple scattering patterns to produce self-consistent ab initio low resolution models. We generated a model for γSA (χ2 = 1.19) shown in Figure S3A, where different colors indicate the individual domains. Further validation is provided by MONSA pairwise analysis of the scattering data of γSA with γSAΔSH3, γSA expressed with an N-terminal SUMOtag (SUMO-γSA), and a stable complex formed between γSA and the kinase domain of FGFR1 (FGFR1/γSA) where the interaction is centered on the nSH2 domain (Figure S3B). Together, the derived model and additional data (Figure S3) suggest a spatial arrangement for the component domains of γSA where the central lobe of the molecular volume is occupied by the spPH and cSH2 domains, sandwiched between the nSH2 and SH3 domains (Figure 3A, inset).

Given the limited flexibility of γSA suggested by the EOM analysis, we further generated a model of its domain structure in terms of the orientation and position of the different domains. We employed a rigid body docking protocol implemented in the program XPLOR-NIH that can incorporate a variety of experimental data types, including SAXS data and other structural information (Figure 3B). For this procedure we treated the spPH, SH3 and nSH2-cSH2 tandem as rigid entities, assumed that the intervening segments were unrestrained and introduced ambiguous distance restraints between the spPH and cSH2 domains based on our NMR data. We consistently obtained models that give a good fit to the SAXS data for γSA (CRYSOL χ2 0.85, XPLOR-NIH RMSD 0.93) with domain arrangement consistent with the MONSA analysis (Figures 3A and 3B).

Taken together with the relatively narrow line widths in the NMR spectra for the 52 kDa γSA construct (Figures 2 and S2) the model depicted in Figure 3 most likely reflects the dynamically averaged conformation for the protein, and the globular SH3, spPH and tandem nSH2-cSH2 units can explore, in a limited way, different relative orientations with little variation of the overall molecular dimensions.

### γSA and Regulation of PLCγ1

**Figure 4:**
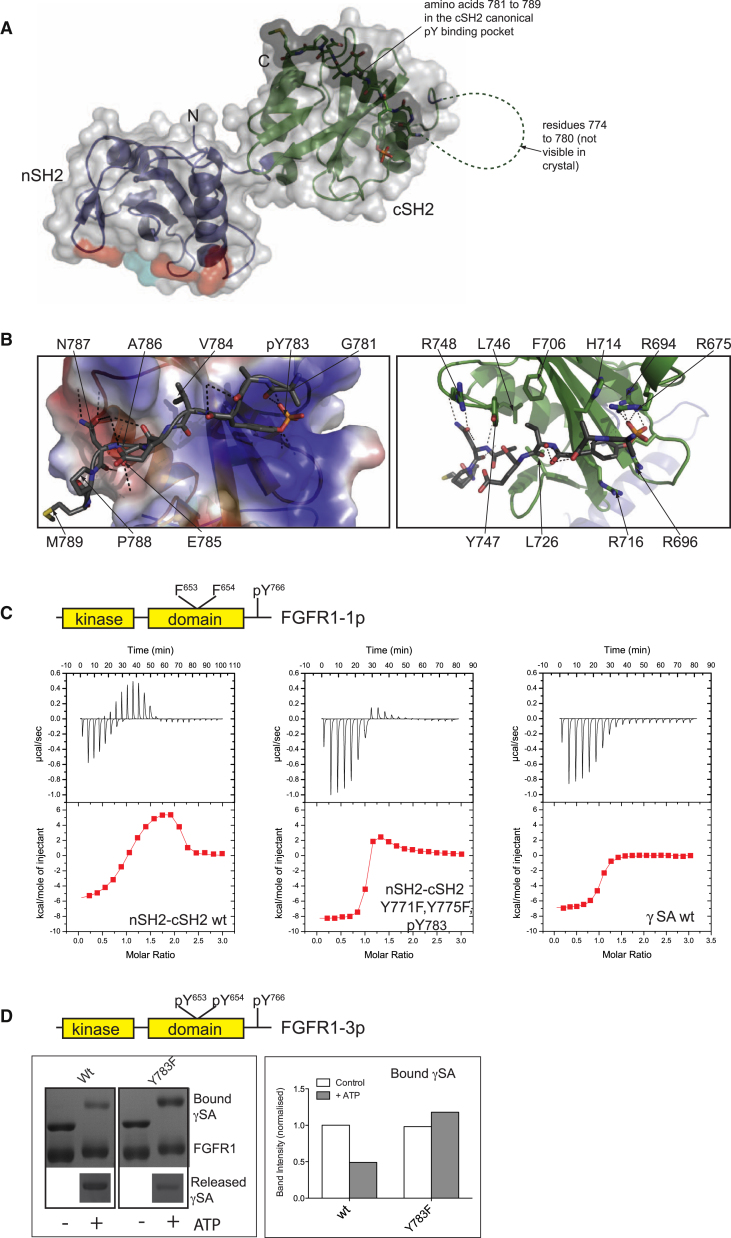
Structure and Properties of the nSH2-cSH2 Tandem Incorporating p-Y783 Linker (A) Overview of the structure of the nSH2-cSH2^Y771/775F, pY783^ intramolecular complex. Surface topology (shown as translucent gray surfaces) and ribbon representation of the structure show the nSH2 domain in blue and the cSH2 in green. Amino acid residues 781 to 789 are represented as sticks and a darker gray surface topology. Amino acid residues 774 to 780, not visible in the crystal structure, are represented by a dashed loop. The region on the nSH2 surface that forms contacts with the FGFR1 kinase domain pY766 and secondary binding site are shown in red and cyan, respectively. (B) Close up of the interaction interface is viewed from above with important amino acid residues of both the cSH2-SH3 linker (left panel) and cSH2 domain (right panel) represented as ball and sticks. In the left panel, the cSH2 surface is represented by an electrostatic charge distribution and in the right panel as a cartoon representation showing the secondary structure elements. Dashed black lines represent hydrogen bonding and salt bridges. (C) ITC curves for the interaction of FGFR1-1p kinase domain with PLCγ1 nSH2-cSH2^wt^ (left panel), nSH2-cSH2^Y771F, Y775F, pY783^ (middle panel) and SA^wt^ (right panel). The bottom panels show the integrated heats as a function of the molar ratio of titrant to protein in the cell. The data were corrected for the heat of dilution of the titrant and subsequently, fit to either a two-site model (left and middle panel) or a one-site model (right panel). (D) Pull-down analysis of γSA^wt^ and γSA^Y783F^ proteins using immobilized FGFR1-3P in the presence and absence of 10 mM ATP. Proteins remaining in the supernatant after incubation are also shown. The main panel shows proteins stained by colloidal coomassie after separation on SDS-PAGE gels. The right panel shows the quantification of the PLCγ1 proteins that were bound to the immobilized FGFR1-3p after the incubation. See also Figure S5.

**Figure 5:**
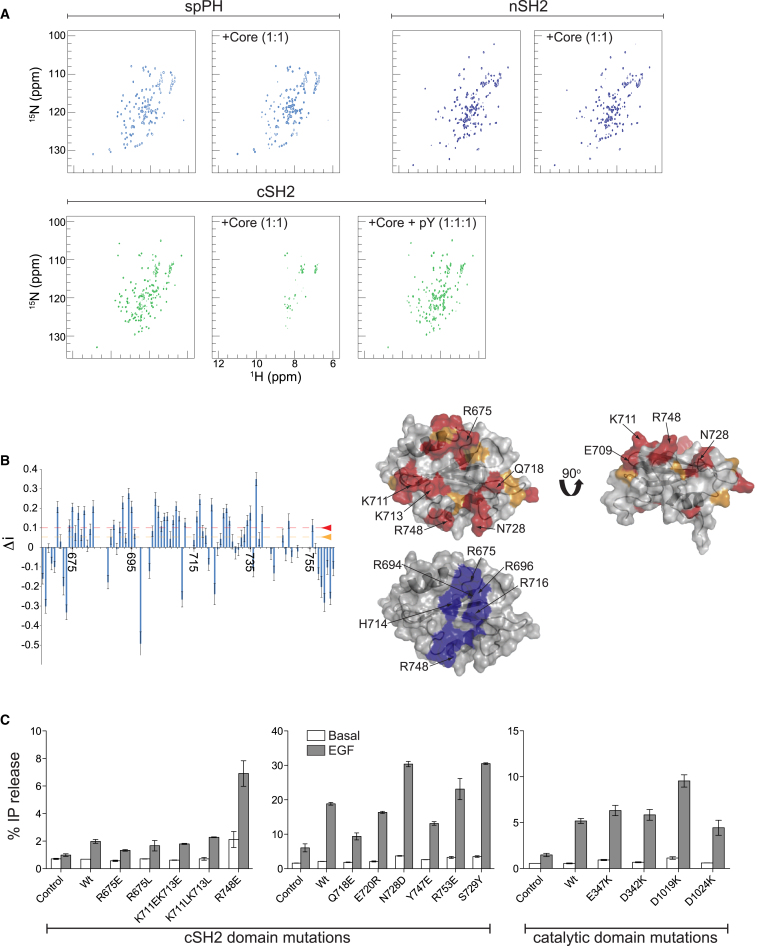
Interaction of γ1SA with the PLC-Core (A) The two-dimensional ^1^H-^15^N HSQC spectra of ^15^N-labeled spPH, nSH2 and cSH2 domain alone and after a 1:1 stoichiometric titration with PLC-core (residues 13−1215, Δ488–933, H335A). For the cSH2 protein a further titration was performed with the PLCγ1-phosphopeptide (NPGFpYVEANPMP) leading to a stoichiometry of the three components of 1:1:1. (B) Residue-specific values of Δ_i_ for ^15^N-labeled cSH2 domain in the presence of 0.4 mole equivalent of unlabeled PLC-core (at this stage of the titration no cross peak has been completely ‘bleached’ from the spectrum as a result of complex formation). Residues with Δ_i_ values between 0.05 and 0.1 were mapped onto a surface representation of the cSH2 domain in orange and those above 0.1 in red. Two-views of the cSH2 domain illustrate that a number of these amino acid residues cluster to a surface. A further surface representation of the cSH2 domain is shown in gray with a number of blue residues which represent the region important for binding the pY783 and surrounding amino acids. (C) The effect of point mutations on basal and stimulated PLCγ1 activity were measured in COS7 cells transfected with pTriEx4-PLCγ1^wt^ and constructs with the indicated point mutations in cSH2 (left and middle panel) and PLC-core (right panel). SD is represented by error bars. See also Figure S6.

Previous studies of PLCγ1 pointed to marked differences in the binding properties of the two SH2 domains (Bae et al., 2009; Gresset et al., 2010). Using an NMR approach, we found that the nSH2 domain is preferentially involved in the binding of receptor-derived phosphopeptides while the cSH2 domain has preference for the phosphopeptide incorporating the critical PLCγ1 phosphorylation site (pY783) in the cSH2-SH3 linker (Figure S5). To analyze the structural and functional implications of Y783 phosphorylation and the potential for intramolecular binding to cSH2, we obtained the crystal structure of a construct incorporating nSH2-cSH2^Y771F,Y775F^ (545–790) following phosphorylation on Y783 (Figure 4A; Table 1). The structure of the tandem protein revealed an intramolecular interaction between the cSH2 canonical pY-peptide binding site and residues ^781^GFpYVEANPM^790^ (Figure 4A). Residues 774 to 780 are not visible in the electron density map suggesting that this part of the linker remains disordered. To orient the FGFR1 binding site on the tandem domain, we have indicated surfaces on the nSH2 corresponding to parts of the FGFR1 canonical and secondary binding sites (Bae et al., 2009; Figure 4A). The structure shows that the cSH2 domain makes an essentially canonical binding interaction with the extended linker in which the pY783 side chain makes multiple interactions with three arginine (R675, 694, and 696) side chains in the conserved pY-binding pocket (Figure 4B). The side chains of residues pY+1 (V784) and pY+3 (A786) are directed into shallower, hydrophobic pockets (formed by cSH2 residues F706, L726, L746, and Y747). The side chains of V784, A786, and P788 all make van der Waals contacts with the cSH2 domain, burying ∼620 Å^2^ of solvent accessible surface. Intriguingly, some less defined density is also observed in the peptide-binding groove for cystals obtained for the nonphosphorylated tandem nSH2-cSH2 protein, suggesting that in the absence of other interactions that might take place within the full-length PLCγ1 (see further, Figure 5) the region of the cSH2-SH3 linker has certain affinity in cis for the pY peptide binding site even in the absence of Y783 phosphorylation.

Although the structure of the cSH2 domain is not affected upon pY783 binding, a further, more detailed comparison of the crystal structure of the phosphorylated nSH2-cSH2 protein with the nonphospho (apo) construct in the same crystal form shows general similarity with some subtle differences in angle and distance between the domains (not shown).

To test the functional implications of this structural integration of the nSH2 and cSH2 domains and changes that accompany binding of pY783 (and surrounding residues), we compared nonphospho and phospho forms of the tandem nSH2-cSH2 for their ability to interact with FGFR1. Isothermal titration calorimetry (ITC) was used to determine the relative affinity of tandem nSH2-cSH2 constructs for FGFR1-1p (pY^766^, binding site for PLCγ1; Table 2; Figure 4C). For wild-type nSH2-cSH2 protein, biphasic binding isotherm was obtained that could be fitted to a two-site binding model. The higher affinity component (attributed to nSH2 binding) exhibits exothermic behavior and a K_D_ of 5 ± 1 nM, while the lower affinity (binding to cSH2) shows endothermic behavior and a K_D_ of 81 ± 16 nM. Measurements performed with nSH2-cSH2^Y771F,Y775F^ tandem phosphorylated to a high level at Y783 show a strikingly different binding isotherm. In this case, a dominant equimolar interaction attributed to interaction of FGFR1-1p with the nSH2 domain is maintained. However, the second binding phase is highly suppressed due to occupancy in *cis* of the canonical pY-binding site on the cSH2 by the pY783-linker region. Interestingly, in this latter instance the binding of FGFR1-1p to the nSH2 domain (K_D_ 44 ± 11 nM) is nine-times weaker than in the case of the non-phosphorylated tandem construct.

Similar measurements were conducted with the γSA and FGFR1-1p (Table 2; Figure 4C). The finding that the binding isotherm has only a single component suggests that within the γSA only the nSH2 domain is available for interaction with FGFR1-1p. Furthermore, attempts to use ITC to analyze binding of phospho-γSA to FGFR1 suggest that similar to the case with the tandem SH2 protein, Y783 phosphorylation reduces the affinity (data not shown). To overcome a limitation resulting from nonhomogeneous in vitro phosphorylation of γSA, we used constructs incorporating a Y783F point mutation and analyzed association with immobilized FGFR1-3p (pY766, pY653, pY654) under conditions where proteins are either nonphosphorylated or phosphorylated (Figure 4D). Using this approach, we found that the otherwise robust association of FGFR1-3p with γSA is reduced following phosphorylation of the wild-type protein, whereas a similar reduction was not observed for the Y783F variant.

Together, the data shown in Figure 4 suggest the possibility of a previously unidentified consequence of the phosphorylation of Y783 and its interaction with the cSH2 binding pocket, namely, a weakening of the affinity between the γSA and FGFR1. In contrast, the link between Y783 phosphorylation and an increase in PLC activity is strongly supported by several lines of experimental evidence reported previously (Suh et al., 2008) as well as by our findings in the context of stimulation by FGFb (Figure 1). To provide a molecular mechanism to rationalise this link, we extended our experiments to include a multidomain construct of PLCγ1 incorporating the PH, EF-hand, catalytic and C2 domains (PLC-core).

Our previous analysis of PLCγ variants (Everett et al., 2011) and data from others (Gresset et al., 2010), demonstrates that a deletion of the cSH2 domain and, under some conditions of the spPH domain, results in an increase in PLC activity. Despite this extensive analysis of PLC activity of various deletion variants, direct binding experiments between domains within the γSA and the PLC-core have not been reported. NMR titration experiments performed with ^15^N-labeled cSH2 domain, nSH2 domain or spPH domain and increasing concentrations of unlabeled PLC-core protein, demonstrate that changes in the NMR spectrum were only observed for the cSH2 domain (Figure 5A). Furthermore, addition of a PLCγ1-derived phosphopeptide from the cSH2-SH3 linker (^779^NPGFpYVEANPMP^790^) completely reversed the broadening of cross peaks in the spectrum. Importantly, analysis of the spectrum at different titration points identified chemical shift changes for residues positioned on a region of the surface of the cSH2 domain that partly overlaps with the phosphopeptide-binding groove (Figure 5B). Both NMR experiments and ITC measurements are consistent with a K_D_ value of about 25 μM for the interaction of these proteins in *trans*, and the observation from the ITC measurements of weak endothermic behavior further suggests the involvement of electrostatic contacts (Table 2). Based on this information we selected a number of residues for mutagenesis and subsequently analyzed their impact on PLCγ activity and affinity between the cSH2 domain and the PLC-core.

Among a number of charge reversal mutations for the cSH2 domain, R748E and R753E have a marked effect on PLC activity in cells, showing higher basal activity and enhanced activation, and loss of a measurable interaction between the cSH2 domain and the PLC-core in vitro (Figures 5C and S6A; Table 2). Two other mutations, N728D and S729Y (the latter based on the PLCγ1 equivalent of a disease linked-mutation in PLCγ2), have a similar effect. Our previous studies of the PLC-core that identified a cluster of negative residues near the active site opening and some specific residues in this region as important for low basal PLC activity (Everett et al., 2009), suggest that this region could be involved in an auto-inhibitory interaction with the cSH2 domain. Mutations in this region of the PLC catalytic domain reveal that D1019K and, to a lesser degree, E347K replacements affect the basal activity and level of activation of PLCγ1 (Figures 5C and S6B). Remarkably, the D1019K PLC-core protein completely loses its ability to interact with the cSH2 domain (Table 2). Thus, mutagenesis experiments and NMR titration data suggest that the cSH2 domain from the γSA solely interacts with the PLC-core and forms the autoinhibitory interface that involves residues at the active site opening. The titration experiments with the phosphopeptide corresponding to the cSH2-SH3 linker further imply that this interaction can be released following Y783 phosphorylation and intramolecular association with the cSH2 domain.

### Mechanistic Implications

**Figure 6:**
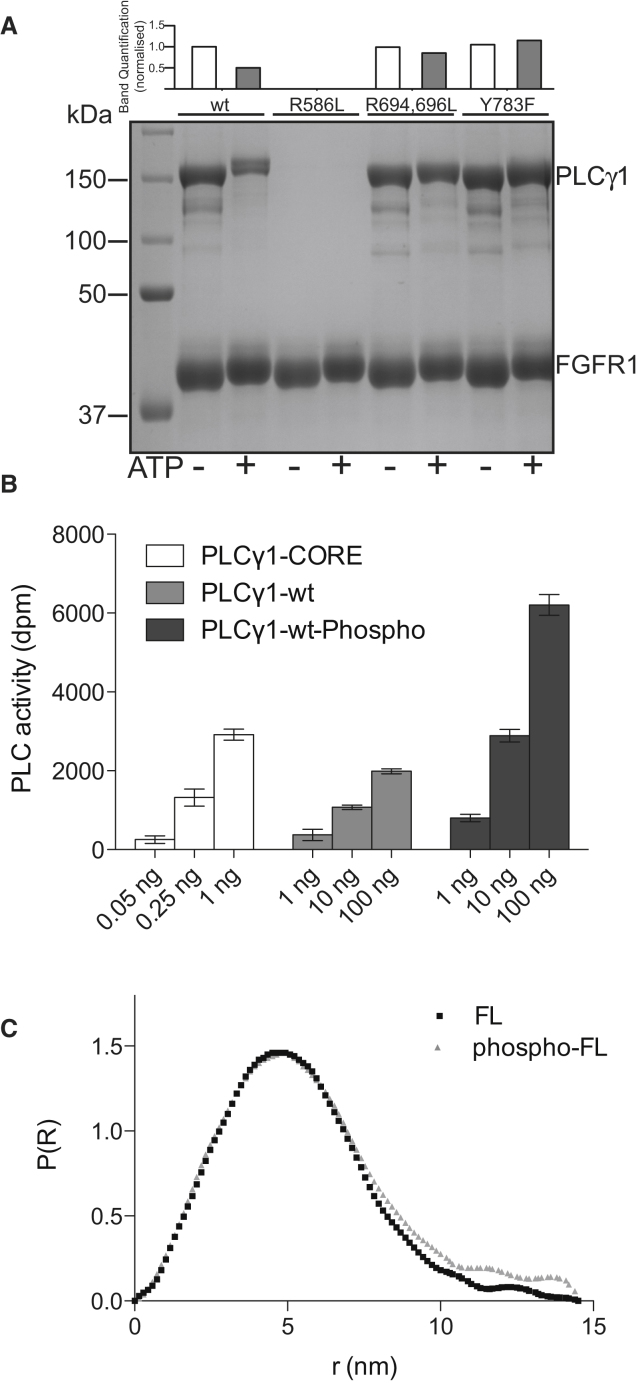
Analysis of Full-Length PLCγ1 (A) Pull-down analysis of full-length PLCγ1^wt^ proteins and the inactivating mutants for nSH2 (R586L), cSH2 (R694L, R696L) or phosphorylation (Y783F) using immobilized FGFR1-3P in the presence and absence of 10 mM ATP. The main panel shows proteins stained by colloidal coomassie after separation on SDS-PAGE gels. The top panel shows the quantification of the PLCγ1 proteins that were bound to the immobilized FGFR1-3p after the incubation. (B) In vitro reconstitution activity assay of purified PLCγ1-core (13–1215, Δ488–933), PLCγ1 full-length and phospho-PLCγ1 (in vitro FGFR1 phosphorylated) was performed at indicated protein concentrations. SD is represented by error bars. (C) Comparison of SAXS data obtained for the unphosphorylated (red) and phosphorylated (blue) full-length PLCγ1; to obtain proteins suitable for SAXS, multi Y to F variant of PLCγ1 was used.

The findings described in Figures 4 and 5 are based on experiments that focus on the γSA; they suggest that the main effects of PLCγ1 phosphorylation by FGFR1 are a weaker interaction between the two proteins and the release of auto-inhibition in PLCγ1. To further establish that these findings are relevant to *holo*-PLCγ1, we analyzed the interaction of the intact enzyme with FGFR1, the activity of PLCγ1 following phosphorylation and the potential for conformational rearrangement within the full-length PLCγ1 molecule (Figure 6).

Using FGFR1-3p and the full-length PLCγ1 protein in the pull down assay described in Figure 4D, we compared the wild-type PLCγ1 protein with variants lacking either a functional nSH2 (R586L) domain, cSH2 (R694L, R696L) domain, or the Y783 phosphorylation site (Y783F; Figure 6A). As demonstrated for the tandem nSH2-cSH2 and γSA (Figures 4C and 4D), the association of wild-type PLCγ1 protein with FGFR1 is greatly decreased following phosphorylation. Furthermore, we could also demonstrate that only a functional nSH2 domain is required for the initial binding to FGFR1 (Figure 6A). This finding is consistent with the data showing a single binding site for FGFR1 within the isolated γSA (Figure 4C). In contrast, the mutations in the cSH2 domain or removal of the Y783 phosphorylation site do not affect initial binding to FGFR1 but have the distinct, common effect of preserving the PLCγ1/FGFR1 complex under conditions that allow protein phosphorylation (Figure 6A). These findings are in full agreement with the intramolecular interaction between pY783 and the cSH2 domain and subsequent functional consequences affecting the nSH2-cSH2 unit (i.e., reduced binding to FGFR1) proposed above.

Our data showing that the nSH2 domain is required for initial binding to FGFR1 (Figures 4 and 6A) while an intramolecular interaction between pY783 and the cSH2 domain effects phospholipase activation (Figure 5) also suggest that phosphorylated PLCγ1 retains high enzyme activity without the requirement for sustained binding to FGFR1; this scenario has not been reported in previous work that instead simply linked PLCγ1 phosphorylation to its activation (Suh et al., 2008). Following phosphorylation of full-length PLCγ1 by FGFR1, we were able to isolate a phospho-enriched form of PLCγ1. Subsequent measurements of PLC activity in vitro clearly show that this phosphorylated PLCγ1 has higher PLC activity although not as high as for PLC-core (Figure 6B).

Based on the data related to the mechanism of overcoming intramolecular inhibition (Figure 5), it might well be expected that PLCγ1 phosphorylation is accompanied by a conformational change. Small angle X-ray scattering measurements of phospho- and nonphospho- full-length PLCγ1 support this possibility: the scattering-derived P(r) distance distribution suggests that phosphorylation results in a less compact overall shape (Figure 6C; Table S1). Unfortunately, due to the complexity of the full-length PLCγ1 it was not yet possible to unambiguously model this change in terms of a three-dimensional (3D) model of the intact enzyme.

**Figure 7:**
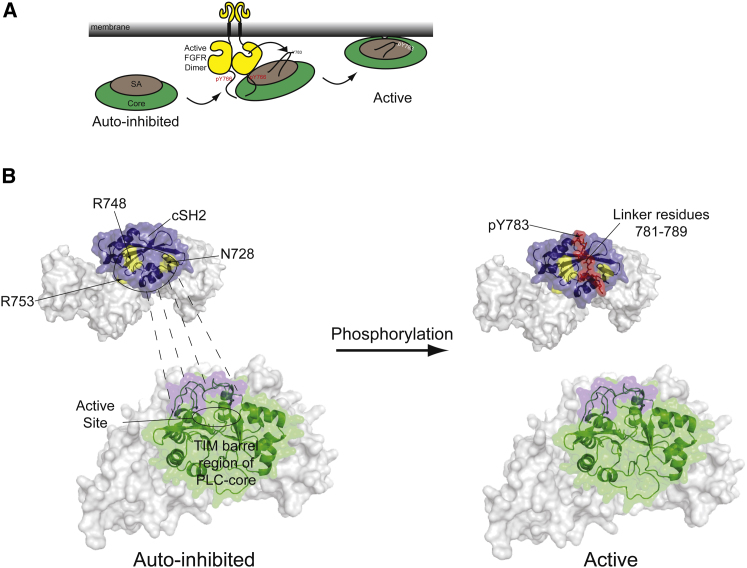
Global and Molecular Models for PLCγ Activation and Signaling Deregulation (A) The two-step global model illustrating activation of PLCγ through recruitment and phosphorylation by receptor tyrosine kinases and the subsequent release and membrane binding. (B) An illustrative summary of the molecular mechanism of autoinhibition and phosphorylation induced activation in PLCγ. The γSA is shown as a gray envelope with the cSH2 region in blue. Amino acid residues in PLCγ1 shown to be important for autoinhibition are colored in yellow and labeled. Similarly, the PLC-core is shown as a gray envelope with the catalytic domain (containing the active site) colored green. The underlying cartoon representation of this region is based on homology to the PLCδ1, PLCβ2 and PLCβ3 PLC-core structures. The surface of the PLC-core above the active site is colored purple and is suggested to interact with the cSH2 domain in the inactive state. Phosphorylation of PLCγ1 causes the intramolecular binding to the cSH2 domain of pY783 and surrounding amino acid residues (red). This interrupts the interaction of the PLC-core with the cSH2 domain leading to the active site gaining access to the membrane and therefore enzyme activation. Most mutations found in immune disorders map to cSH2 domain (blue) and the surface above the active site (purple).

Taken together, the data obtained using the full-length protein support a global model illustrated in Figure 7A. According to this model, the strong interaction between FGFR1 and PLCγ1 could be transient and a more loosely interacting or released, phosphorylated PLCγ1, with high enzyme activity, could make direct interaction with the membrane and gain access to resident PIP_2_ phospholipid substrates. This could allow FGFR to processively phosphorylate many PLCγ molecules. Importantly, our structural insights reveal the molecular mechanism that could underpin this global activation mechanism. Notably, in addition to our finding that the tandem SH2 domains effectively form a coupled structural unit and could interact functionally, we also describe the molecular mechanism of the release of auto-inhibition. As depicted in Figure 7B, the autoinhibition imposed on the PLC catalytic domain by the cSH2 domain is released by direct competition between the segment of the cSH2-SH3 linker surrounding pY783 and the surface on the PLC-core binding to overlapping surfaces on the cSH2 domain.

## Discussion

Previous insights into the regulation of PLCγ have confirmed that, as for other PLC families, PLCγ is autoinhibited by intramolecular constraints, the release of which lead to enzyme activation (Everett et al., 2011; Gresset et al., 2010). However, a limitation of these studies is a lack of understanding of the 3D organization of these complex proteins, in particular of the regulatory region, which would allow a deeper and more comprehensive analysis of the molecular mechanisms. Using a combination of NMR, SAXS, X-ray crystallography, and biochemical techniques, we have been able to define the architecture of this region and its interaction with the PLC-core to generate an overall picture of how PLCγ enzymes are regulated.

We demonstrate that among four domains present in the γSA, the cSH2 domain and its C-terminal linker provide a focus for most interdomain interactions both within the γSA and between the γSA and the PLC-core. Restraints are imposed on the cSH2 domain by an apparently inflexible linker to the nSH2 domain. Indeed the two SH2 domains could be thought of as forming a single supramodule as defined by the NMR-derived rotational correlation time (Figure 2C). In contrast, our data suggest that the spPH domain and SH3 domain make weaker and more transient interactions with the cSH2 or the linker region, respectively (Figures 2D and 2E). Importantly, only the cSH2 domain appears to be involved in binding to the PLC-core and based on interactions using isolated fragments in *trans*, intramolecular interactions in *cis* are likely to be quite strong (Figure 5). This central position of the cSH2 domain within PLCγ enzymes is in full agreement with the findings that the deletion of the cSH2 domain in PLCγ1 and PLCγ2 results in release of auto-inhibition and constitutive PLC activity (Everett et al., 2011; Gresset et al., 2010). The relative flexibility and weak interactions that involve the spPH and SH3 domains may allow better adaptability to different interaction partners or simultaneous recognition of multiple binding sites involved in the regulation of PLCγ. Such arrangements could, for example in the case of PLCγ2, facilitate regulation by Rac that binds directly to the spPH domain (Bunney et al., 2009). This in turn could impact on the auto-inhibitory role of the cSH2 domain on PLC activity in its own right or in synergy with other inputs. Similarly, the relative independence of the SH3 domain would allow interactions that, as suggested for cCbl, are not involved with auto-inhibition and enzyme activation but instead could be linked to downregulation of the protein (Tvorogov and Carpenter, 2002). In the context of the activation by FGFR1, and more generally for the activation by tyrosine kinase receptors, the tight structural and functional link between the nSH2 and cSH2 domains is critical (Figure 7).

Studies in murine models (Abe et al., 2011; Yu et al., 2005) and recent genetic studies of patients and their families (Ombrello et al., 2012) have demonstrated a link between dominantly inherited complex immune disorders and gain-of-function mutations in PLCγ2. Based on our initial characterization of these disease-linked variants, the PLCγ2 mutations and corresponding mutations in PLCγ1 seem to compromise the auto-inhibitory mechanism (Everett et al., 2009). Indeed, two distinct deletion variants found in PLAID patients lack portions of the cSH2 domain and likely disrupt proper folding of this domain and almost completely remove the auto-inhibitory surface. Consistent with this, PLAID mutants of PLCγ2 appears to be constitutively active (Ombrello et al., 2012). Interestingly, a point mutation in the cSH2 domain (S707Y in PLCγ2), found in a family characterized with another set of immune disorder manifestations (Zhou et al., 2012), corresponds to S729Y in PLCγ1. As shown (Table 2; Figure 5C), this mutation reduces the interaction between the cSH2 domain and the PLC-core and results in an increase in basal activity (albeit less pronounced than for PLAID deletions) and a notable enhancement of PLC activity following stimulation. This is similar to the effects of other point mutations in PLCγ2 found in murine models (Ali5 [D993G] and Ali14 [Y495C] loci corresponding to D1019 and Y509 in PLCγ1, respectively; Everett et al., 2009). Ali5, located in the PLC-core, corresponds to a residue mutated here to a oppositely charged amino acid (D1019K). This mutation has the most striking effect on both, increasing basal enzyme activity and direct binding between the cSH2 and PLC-core (Table 2; Figures 5C and S6B). Intriguingly, the Ali14 mutation locus appears to be at the interface of the spPH and cSH2 domains, mapped here by NMR (Figure 2D); similar, activating mutations were independently reported in PLCγ1 (DeBell et al., 2007). These results are therefore consistent with the possibility that the spPH-cSH2 interaction could indirectly affect auto-inhibition.

Although many signaling enzymes, including tyrosine phosphatases, tyrosine kinases, lipid kinases and phospholipases, are constructed with structurally homologous modular domains, it is clear that distinct mechanisms must have evolved to control their activities. The diversity of these mechanisms is also clear from comparison of different Src, Abl, Syk and Fes family kinases. For example, several structures have shown that SH2 domains of tyrosine kinases such as Src, Abl, and Zap70 have an ability to suppress kinase activity through intramolecular interactions (Filippakopoulos et al., 2009). Recently, the structure of the SH2 domain linked to the kinase domain of Fes revealed that the SH2 domain can also stabilize the active kinase conformation, in this case by direct interactions with the regulatory αC helix; this interaction is further stabilized by ligand binding to the SH2 domain (Filippakopoulos et al., 2008). In such cases, kinase activation could be closely coupled to substrate recognition through cooperative SH2-kinase-substrate interactions. In the lipid kinase PI3Kβ two SH2 domains bind to the regulatory region, that encloses the catalytic and activation loop, and impose autoinhibition (Zhang et al., 2011). Nevertheless, despite the diversity, what seems to be a main common theme for structurally defined proteins incorporating SH2 domains is that SH2 domains involve allosteric regulation of the catalytic function. One notable exception is the nSH2 domain of SHP-2 tyrosine phosphatase that binds to the phosphatase domain and directly blocks its active site (Hof et al., 1998). It is also usually the case that the same SH2 domain that interacts with an catalytic domain also binds directly to a pY motif within regulatory proteins; examples include SH2 domains from Abl, Fes, SHP-2, and PI3Kβ (Filippakopoulos et al., 2009; Hof et al., 1998; Zhang et al., 2011). The mechanism for PLCγ has some quite different, distinct characteristics shown here in the context of regulation via FGFR1. Notably, the function of the binding to FGFR1 and the intramolecular interaction with the PLC-core are distributed between two SH2 domains. The nSH2 interacts with upstream receptors (showing no conformational change upon binding) and its main function is to recruit and facilitate phosphorylation in *trans* (Figures 4 and 6). The cSH2, in contrast, makes contacts with the PLC-core and recognizes an intramolecular pY motif rather than a receptor site. It also seems that a relatively small change in the tandem cSH2-nSH2—induced upon pY783 binding to cSH2—is allosterically relayed to the nSH2 domain reducing the affinity for FGFR1 but with no corresponding allosteric effect on PLC activity. Instead, it is likely that the cSH2 acts as a lid with precise contacts with the PLC-core that becomes removed by interaction with the pY783 motif (Figure 5). This mode of regulation (Figure 7) is consistent with the observations that the PLC catalytic domain, a TIM barrel, forms an effectively rigid structure, not influenced by substrate binding (Bunney and Katan, 2011). This is also in agreement with the regulatory models proposed for other PLC families, which have in common that autoinhibition is mainly brought about by active site occlusion, and in the case of PLCβ2/3 and PLCδ1 enzymes, by more flexible and mobile elements (Hicks et al., 2008).

## Experimental Procedures

### Cloning, Expression, and Purification of Recombinant Proteins

Genes or their fragments to be expressed were cloned into pOPINS (Oxford Protein Production Facility), pTriEx4 or pTriEx6 (Novagen). *E. coli* strain C41(DE3; Lucigen) or the mammalian cell line Freestyle 293F (Invitrogen) were used following manufacturer's instructions. Purification was performed using Ni^2+^ chelating chromatography with cleavable His-tags. An expanded set of procedures can be found in the Supplemental Information.

### Crystallography, NMR, and SAXS Measurements

Proteins were prepared as outlined in the Supplemental Information and phosphorylated by FGFR1 where indicated. Crystals of both apo and phosphorylated nSH2-cSH2 were prepared by the hanging drop vapor diffusion method. Conditions and further details can be found in the Supplemental Information. Final images were created using the program PYMOL.

All NMR experiments were carried out at 25°C on Varian INOVA or Bruker AVANCE spectrometers operating at 14.1 T or 16.5 T equipped with a cryoprobe. Details of data processing, semiquantitative analysis of cross-peak broadening and molecular modeling are given in the Supplemental Information.

Synchrotron SAXS data were collected on the EMBL X33 camera with a Pilatus detector on the storage ring DORIS III (Deutsches Elektronen-Synchrotron, Hamburg, Germany) and ESRF ID14-3 camera with a Pilatus detector (ESRF, Grenoble, France). Details of data analysis with DAMMIN and MONSA software packages are supplied in the Supplemental Information.

### Functional Assays

ITC experiments were carried out with either a Microcal VP-ITC machine (as described in Bunney et al., 2006, 2009) or an iTC_200_ system (MicroCal). Experimental details are supplied in either figure legends or the Supplemental Information.

Pull-down assays with immobilized FGFR1 and various constructs of PLCγ1 were performed with Streptactin Macropore beads following manufacturer's instructions. Specific experimental conditions can be found in the Supplemental Information.

Assays of PLC activity in COS7 and PAE cells were carried out as outlined in (Bunney et al., 2009; Everett et al., 2009) with experimental differences outlined in the Supplemental Information. Details of PLC activity measured in vitro, described in (Bunney et al., 2009), and other assays associated with measuring protein function can be found in the Supplemental Information.

## Figures and Tables

**Table 1:** X-Ray Crystal Structures Data Collection and Refinement Statistics: Molecular Replacement

	nSH2-cSH2wt	nSH2-cSH2^Y771F,'Y775F,'pY783^
**Data Collection**		

Space group	P21221	P1

**Cell dimensions**		

a, *b, c* (Å)	53.7 59.6 76.9	54.9 59.2 79.2
α, β, γ (°)	90.0, 90.0, 90.0	90.0, 90.0, 90.0
Resolution (Å)	44.06–2.4 (2.68–2.4)	47.42–2.8 (2.95–2.8)
*R*_sym_ or *R*_merge_	0.045 (0.974)	0.099 (0.762)
*R*_*meas*_P1/P21221	0.048/0.051	0.139/0.161
//σ/	17.4 (1.7)	6.2 (1.1)
Completeness (%)	96.2 (96.2)	92.1 (96.4)
Redundancy	4.5 (4.5)	2.0 (2.0)

**Refinement**		

Resolution (Å)	2.4	2.8
Total No.	9,496	22,717

**reflections**		

*R*_work_/*R*_free_	0.219/0.295	0.184/0.249

**No. atoms**		

Protein	1,802	7,492
Ligand/ion	0	0
Water	32	249

***B*-factors**		

Protein	79.829	54.774
Ligand/ion	–	–
Water	67.11	34.125

**Rmsds**		

Bond lengths (Å)	0.010	0.010
Bond angles (°)	1.11	1.33

One crystal was used for each data collection. Values in parentheses are for highest-resolution shell. R_free_ values are 5% of total number of reflections.

**Table 2:** Thermodynamic Quantities for the Interaction PLCγ 1 SH2 Domains with FGFR1-1p and the Binding of PLC-Core to the PLCγ1 cSH2 Domain

Protein					
Cell	Syringe	n	K_D_ (μM)	ΔH° (cal mol^−1^)	ΔS (cal mol^−1^ K^−1^)
nSH2cSH2^WT^	FGFR1-1p				
First Binding Event		0.98 ± 0.01	0.005 ± 0.001	−6,501 ± 183	16.1
Second Binding Event		1.08 ± 0.02	0.081 ± 0.016	7,116 ± 230	56.3
nSH2cSH2^pY783^	FGFR1-1p				
First Binding Event		0.998 ± 0.009	0.044 ± 0.011	−8,307 ± 25	5.8
Second Binding Event		0.084 ± 0.11	6.45 ± 0.93	37710 ± 5130	150
γSA^WT^	FGFR1-1p	1.03 ± 0.19	0.185 ± 0.061	−7,093 ± 167	7.02
PLC-Core^H335A^	cSH2^WT^	1.14 ± 0.04	23.9 ± 5.0	586.9 ± 37.1	23.1
PLC-Core^H335A^	cSH2^N728D^	1.17 ± 0.06	49.5 ± 12.3	−1,432 ± 134	14.8
PLC-Core^H335A^	cSH2^S729Y^	1.00 ± 0.04	61.3 ± 8.2	−1,449 ± 97	14.3
PLC-Core^H335A^	cSH2^R748E^	–		NB	NB
PLC-Core^H335A^	cSH2^R753E^	–		NB	NB
PLC-Core^H335A,D1019K^	cSH2^WT^	–		NB	NB

NB, no heat of interaction detected; PLC-Core H335A, PLC inactive variant with high protein yield.

## Accession Numbers

The PDB ID codes for the crystal structures of the nSH2-cSH2 tandem and phospho-nSH2-cSH2 tandem are 4FBN and 4EY0.

## Supplemental Information

Document S1. Figures S1–S6, Table S1, and Supplemental Experimental Procedures
