# Supplementary material for: Structural and Functional Integration of the PLCγ Interaction Domains Critical for Regulatory Mechanisms and Signaling Deregulation
Source: Structure. 2012 Dec 5;20(12):2062–75. doi: 10.1016/j.str.2012.09.005 (PMC3532599; doi:10.1016/j.str.2012.09.005)
Supplement: Document S1. Figures S1–S6, Table S1, and Supplemental Experimental Procedures [file mmc1.pdf]

Structure, Volume *20*

Supplemental Information

Structural and Functional Integration of the PLCγ Interaction Domains Critical for Regulatory Mechanisms and Signaling Deregulation

Tom D. Bunney, Diego Esposito, Corine Mas-Droux, Ekatarina Lamber, Rhona W. Baxendale, Marta Martins, Ambrose Cole, Dmitri Svergun, Paul C. Driscoll, and Matilda Katan

Inventory of Supplemental Information

Figure S1. Summary of PLCγ1 phosphorylation sites and assessment of their importance for activation by FGFR1, related to Figure 1

Figure S2. Overlay of NMR spectra and γSA assignments, related to Figure 2

Figure S3. SAXS data for different constructs and fitting of multiple structures, related to Figure 3

Figure S4. Diagrams illustrating flexibility and range of possible distances between different domains within the γSA, related to Figure 3

Figure S5. Preferences of peptide binding to SH2 domains, related to Figure 4

Figure S6. Further analysis of cSH2 and catalytic domain mutations in COS cells, related to Figure 5

Table S1. Overall parameters of PLC©1 fragments from SAXS data, related to Figures 3 and 6

Supplemental Experimental Procedures

Supplemental References

A

Sequence	Peptide *m/z*	Mass error (ppm)	Phosphorylation site
K.^{179}NMLSQVNYR^{187}.V	602.7624^{2+}	-0.34	Y186
K.^{465}KLAEGSAYEEVPTSMMYSENDISNSIK^{491}.N	1035.7782^{3+}	-3.8	Y472
K.^{466}LAEGSAYEEVPTSMMYSENDISNSIK^{491}.N	993.0814^{3+}	-2.4	Y481
K.^{764}IGTAEPDYGALYEGR^{778}.N	846.3690^{2+}	-0.46	Y771
K.^{764}IGTAEPDYGALYEGR^{778}.N	846.3683^{2+}	-1.3	Y775
R.^{779}NPGFYVEANPMPTFK^{793}.C	896.3933^{2+}	-1.04	Y783
K.^{949}IALELSELVVYCRPVPFDEEK^{969}.I	862.7563^{3+}	-0.72	Y959
R.^{975}ACYRDMSSFPETK^{987}.A	836.3293^{2+}	-1.8	Y977
R.^{1254}YQQPFEDFR^{1262}.I	655.2661^{2+}	0.027	Y1254

B

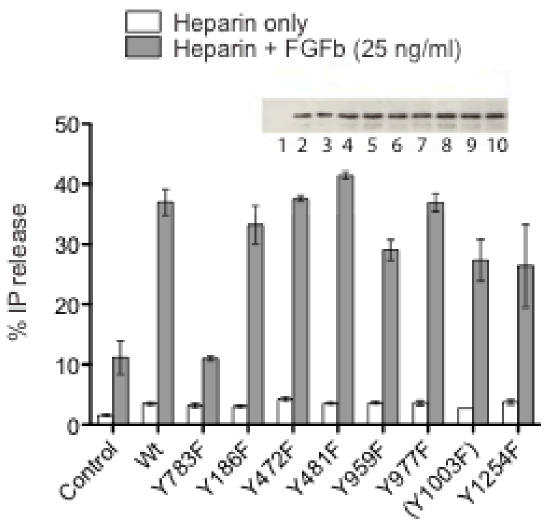

Figure S1. Summary of PLCγ1 phosphorylation sites and assessment of their importance for activation by FGFR1, related to Figure 1

(A) LC-MS/MS phosphopeptide analysis (LTQ Velos Orbitrap mass spectrometer) of PLCγ1 was performed following *in vitro* phosphorylation by FGFR1 kinase domain. Peptides are derived from either trypsin or Lys-C based workflows. Analysis of MS/MS was performed using Proteome Discoverer v1.2, Mascot v2.2. and Scaffold v3.0.

(B) The effect of point mutations on basal and FGFb stimulated PLCγ1 activity were measured in PAE cells transfected with pTriEx4-PLCγ1^{wt} and constructs containing indicated point mutations. Western blotting was used to show equal expression (inset). SD is represented by error bars. Data are representative for three independent experiments.

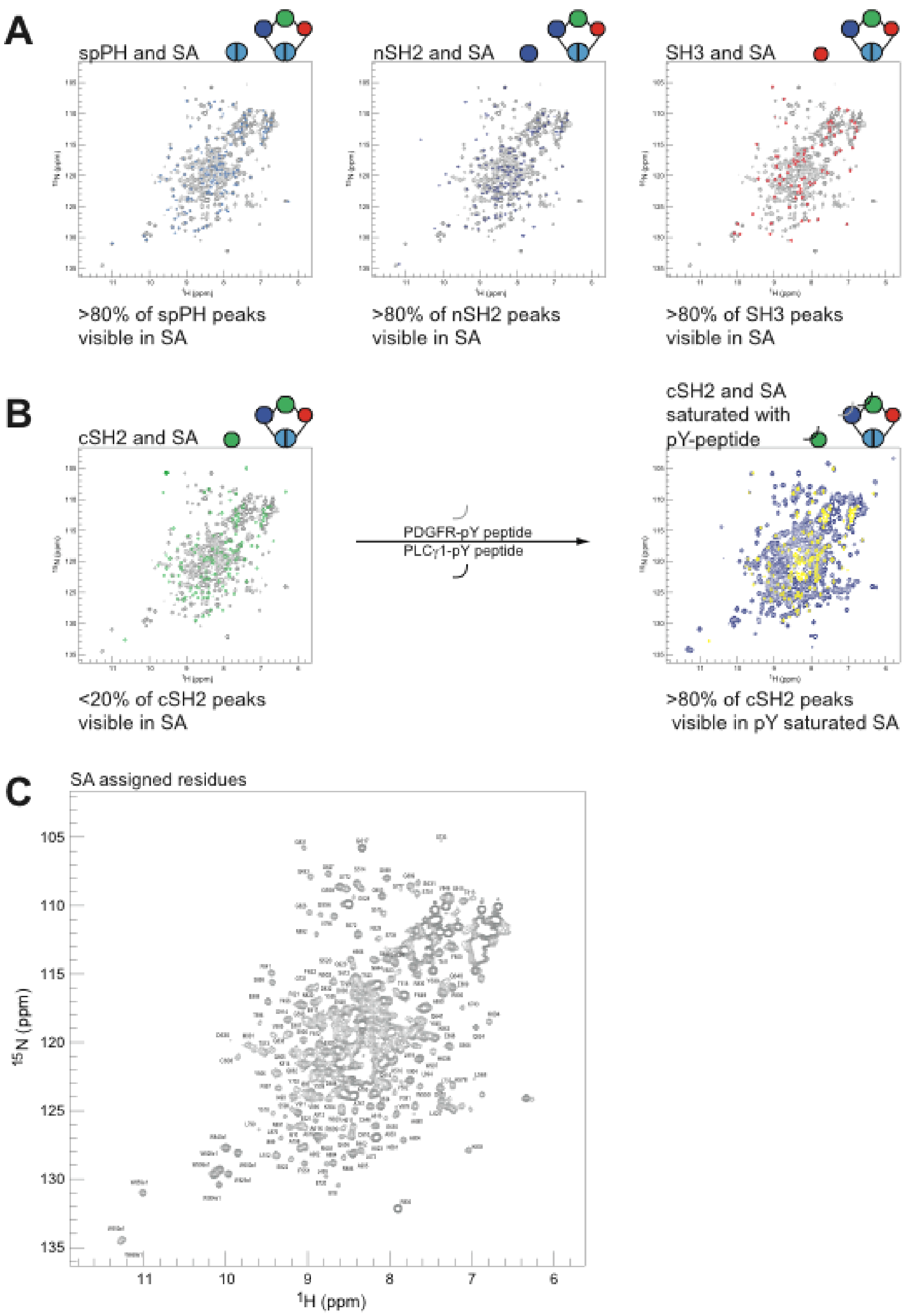

Figure S2. Overlay of NMR spectra and γSA assignments, related to Figure 2

(A) The two-dimensional ^{1}H-^{15}N HSQC spectra of ^{15}N-labelled γSA (grey) overlaid with the spPH (cyan, left), nSH2 (blue, middle) and SH3 (red, right) spectra. (B) The two-dimensional ^{1}H-^{15}N HSQC spectra of ^{15}N-labelled γSA (grey) overlaid with the cSH2 (green) spectra (left). The two-dimensional ^{1}H-^{15}N HSQC spectra of ^{15}N-labelled γSA that was peptide saturated (1:10 stoichiometric excess of NPGFpYVEANPMP (PLCγ1 pY-peptide) and DNDpYYIIPLPDPK (PDGFRβ pY-peptide)) (purple) overlaid with the cSH2 peptide saturated (1:10 stoichiometric excess of NPGFpYVEANPMP (PLCγ1 pY-peptide)) (yellow) spectra (right). (C) The two-dimensional ^{1}H-^{15}N HSQC spectra of ^{15}N-labelled γSA (grey) partially backbone assigned.

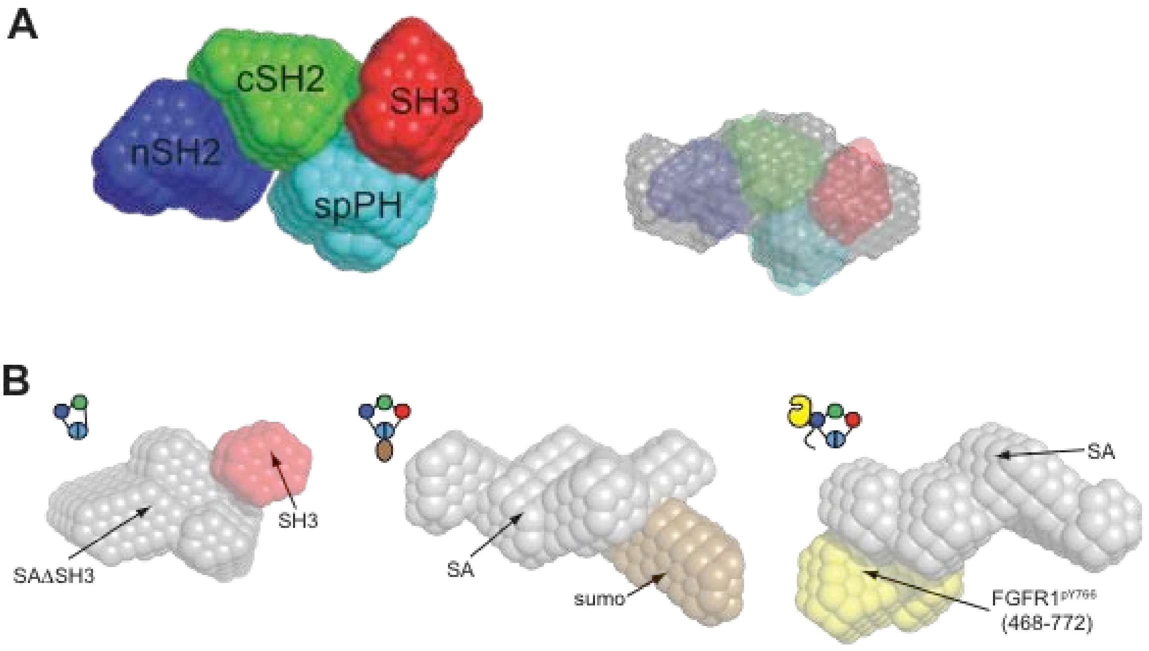

Figure S3. SAXS data for different constructs and fitting of multiple structures, related to Figure 3

(A) Summary model showing the positions of the domains within γSA as predicted by simultaneous 5-fit MONSA modelling. Inset shows the MONSA model overlaid with the γSA envelope produced with DAMMIN. (B) *Ab initio* MONSA models of γSA compared to γSAΔSH3 with the SH3 (red) and γSAΔSH3 (grey) regions shown (left). *Ab initio* MONSA models of γSA compared to γSA-Sumo with the sumo (brown) and γSA (grey) regions shown (middle). *Ab initio* MONSA models of γSA compared to γSA/FGFR1 complex with the FGFR1 (yellow) and γSA (grey) regions shown (right).

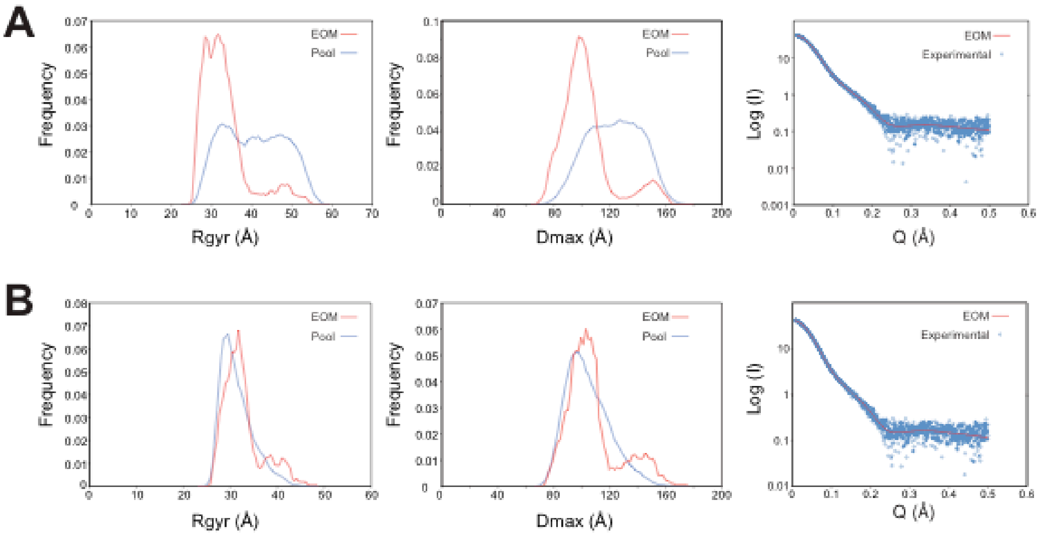

Figure S4. Diagrams illustrating flexibility and range of possible distances between different domains within the γSA, related to Figure 3

Radius of gyration, Dmax distributions and Log of scattered intensity versus Q of the pool (blue line) and selected subsets (red line) and experimental SAXS data (cyan circles) for the Ensemble Optimization Method (EOM) analysis of γSA. Assuming a fully flexible γSA (A), restrained by deduced interface between spPH and cSH2 (B). Restrains imposed by spPH/cSH2 interaction were consistent with the EOM analysis of SAXS data for the spPH-cSH2 construct. However, based on a similar analysis of relevant constructs, SH3/linker interactions suggested by NMR appear to have less impact. In both A and B, the inter-domain linkers have been considered as fully flexible, which can be considered as one extreme scenario of the actual situation.

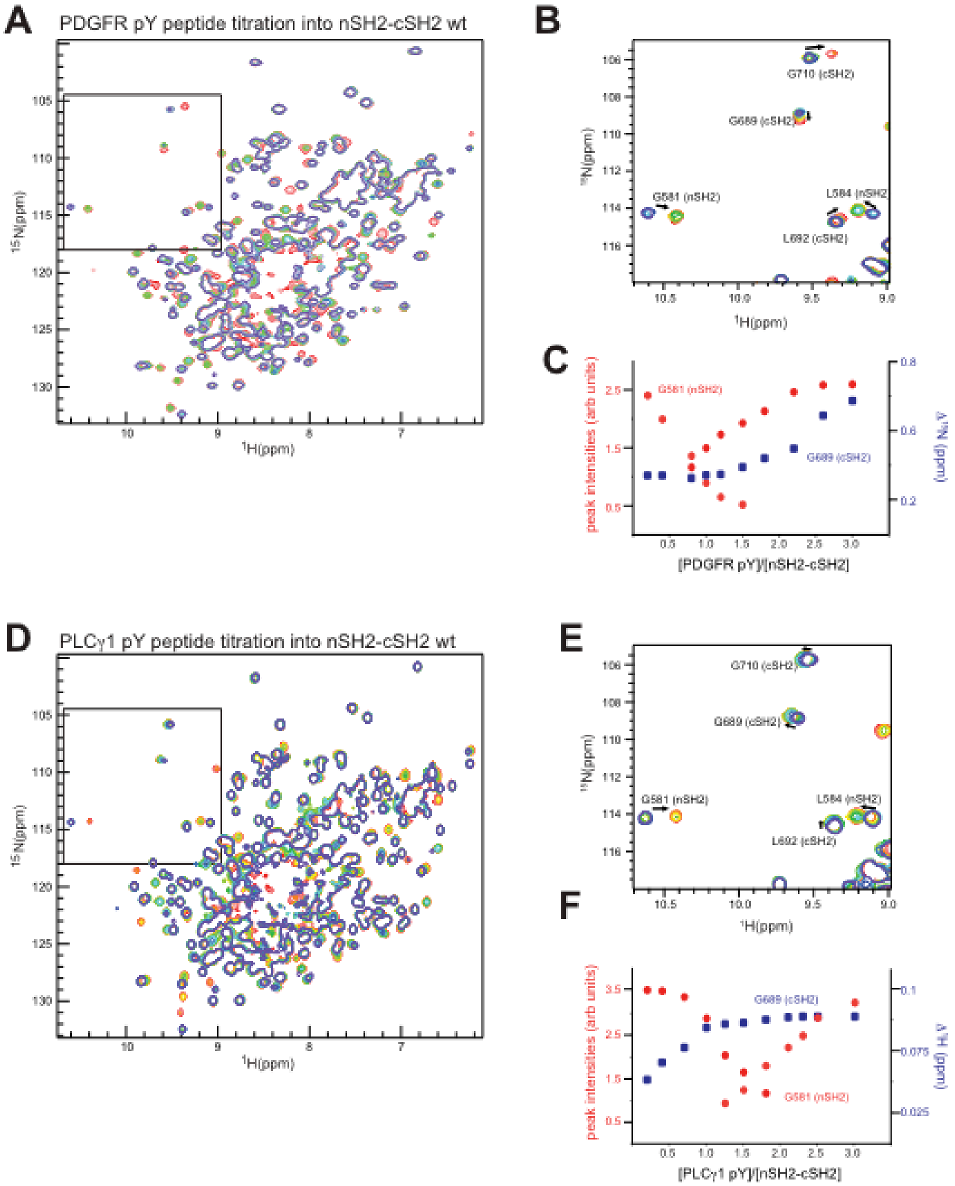

Figure S5. Preferences of peptide binding to SH2 domains, related to Figure 4
(A) NMR titration of nSH2-cSH2^{wt} with PDGFRβ pY-peptide (DNDpYYIIPLPDPK). An overlay of the two-dimensional ^{1}H-^{15}N HSQC spectra of ^{15}N-labelled nSH2-cSH2^{wt} are shown in *purple*, *blue*, *cyan*, *green*, *orange* and *red* at 0, 0.2, 0.6, 1.0, 1.6 and 3.0 equivalents (molar ratio of peptide to protein) respectively. (B) Shows the excerpt of the enclosed region of the spectrum in (A) with assignment of selected peaks from the nSH2 and cSH2 domains. (C) Quantification of either the peak intensities (for nSH2 residues) or the chemical shift perturbations (for cSH2 residues) of selected peaks from (B). (D) NMR titration of nSH2-cSH2^{wt} with PLCγ1 pY-peptide (NPGFpYVEANPMP). An overlay of the two-dimensional ^{1}H-^{15}N HSQC spectra of ^{15}N-labelled nSH2-cSH2^{wt} are shown in *purple*, *blue*, *cyan*, *green*, *yellow*, *orange* and *red* at 0, 0.2, 0.7, 1.0, 1.8, 2.1 and 3.0 equivalents (molar ratio of peptide to protein) respectively. (E) Shows the excerpt of the enclosed region of the spectrum in (D) with assignment of selected peaks from the nSH2 and cSH2 domains. (F) Quantification of either the peak intensities (for nSH2 residues) or the chemical shift perturbations (for cSH2 residues) of selected peaks from (E).

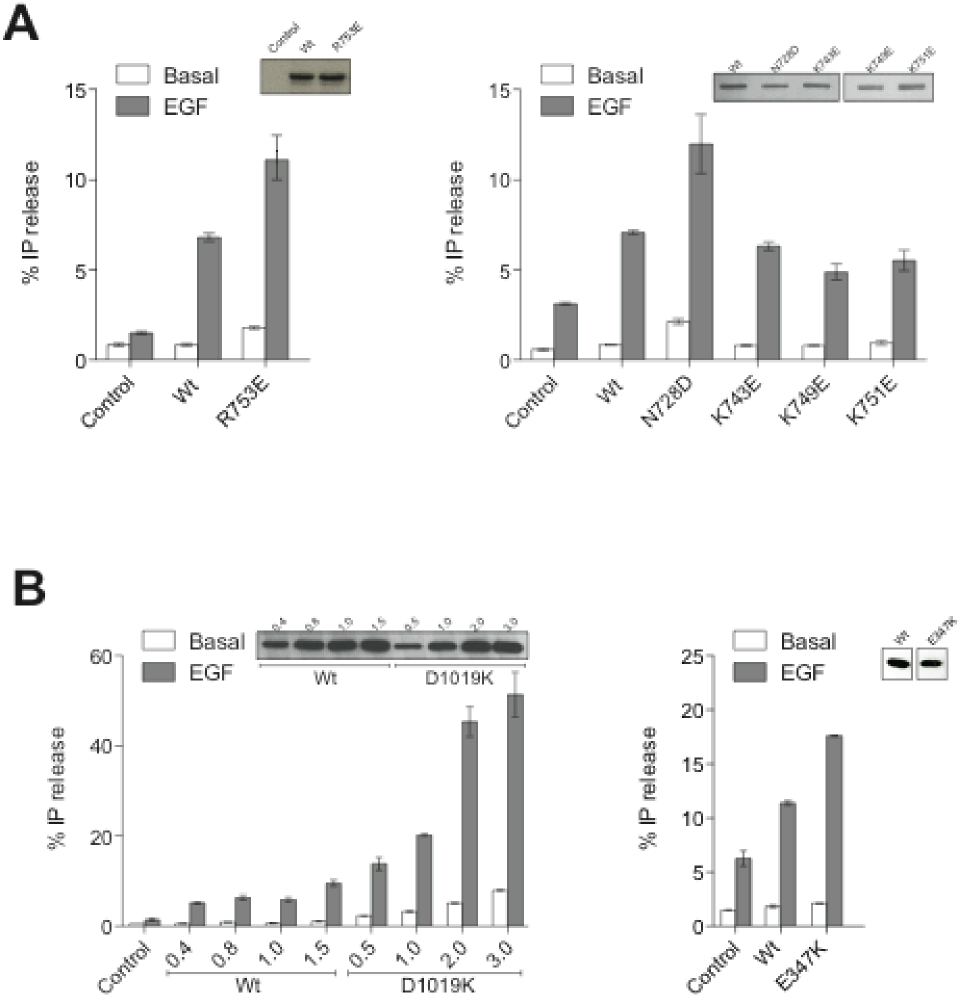

Figure S6. Further analysis of cSH2 and catalytic domain mutations in COS cells, related to Figure 5

The effect of point mutations on basal and EGF stimulated PLCγ1 activity were measured in COS7 cells transfected with pTriEx4-PLCγ1^{wt} and constructs containing indicated point mutations in cSH2 domain (A) and catalytic domain (B). For comparison of wild type with D1019K mutation in the catalytic domain, PLC activity measured for a range of expression levels (plasmid concentrations are indicated) is shown (left panel). Western blotting was used to show protein expression (insets). SD is represented by error bars.

Table S1. Overall parameters of PLCγ1 fragments from SAXS data.

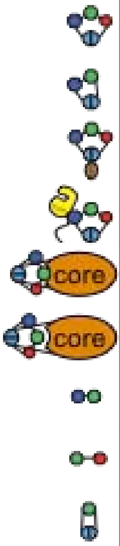

Sample	Theoretical Mass (kDa)	Calculated Mass (kDa)	Rg (nm)	Dmax (nm)	Excluded volume (nm^3)	χ^2
SA	51.4	42	3.2 ± 0.006	10.5 ± 0.5	111 ± 2	1.075
SAΔSH3	44.3	34	2.9 ± 0.005	9.5 ± 0.5	78 ± 2	0.952
SUMO-SA	63.8	54	3.8 ± 0.007	13.0 ± 0.5	185 ±7	1.056
FGFR1/SA	87.6	72	4.0 ± 0.013	14.0 ± 0.5	213 ±5	0.865
FL	154.7	111	4.0 ± 0.015	14.0 ± 0.5	330 ±5	0.974
Phospho-FL	154.7	112	4.3 ± 0.012	14.4 ± 0.5	328 ± 4	1.169
tandem SH2	28.5	21	2.4± 0.008	8.3 ± 0.5	47 ± 3	1.18*
cSH2-SH3	24.1	20	2.5± 0.007	8.6 ± 0.5	46 ± 5	0.991
spPH-cSH2	27.4	21	2.4± 0.007	8.1 ± 0.5	51 ± 4	0.989

*χ^2 values are presented for modelling performed with DAMMIN with the exception of the tandem SH2 domains for which the values are derived from MONSA fitting.

SUPPLEMENTAL EXPERIMENTAL PROCEDURES

Cloning, expression and purification of recombinant proteins

Construction of vectors- Full-length human PLCγ1 was cloned using Gateway technology (Invitrogen) into pDONR207 (Invitrogen) and after sequencing transferred by the LR reaction into a Gateway modified version of pTriEx4 (Novagen). These constructs were used primarily for transfections of COS7, PAE and 293F cells and were expressed with an N-terminal Hisx6 tag followed by an S-tag that was used for protein purification and detection of protein expression in Western blotting respectively. Core constructs of PLCγ1 comprising amino acids 13-1215, Δ488-933, H335A were also cloned with Gateway technology into pTriEx4 (via pDONR207) and used in transfections of 293F suspension cells. In this case an HRV 3c protease recognition sequence was included to allow a native N-terminus (amino acid residue 13) after cleavage. All specific array and smaller constructs were cloned into vector pOPINS (Oxford Protein Production Facility) using In-Fusion technology (Clontech). The exceptions to this were the SH3 domain (791-851, C794S) and spPH domain (488-933, Δ530-864) that were cloned into pTriEx4 using Ek/LIC cloning (Novagen) and incorporated an N-terminal TeV protease recognition sequence.

A synthetic ORF encoding for the human FGFR1 kinase domain (456-774, L457V, Y463F, C488A, Y583F, C584S, Y585F) was prepared by EuroFins MWG. A construct was prepared by splicing PCR consisting of the following elements in order, 2 x StrpII tags, Sumo Star domain, FGFR1 (464-774) and a 10 x His-tag. This construct was cloned into pTriEx6 using 3C/LIC cloning (Novagen). The resulting protein was designated as FGFR1-3p on account of the fact that three tyrosines only

can be phosphorylated in this construct (Y653, 654 and 766) The FGFR1opt (456-774) was also cloned into pOPINS using In-Fusion cloning and the active site tyrosines, 653 and 654, were mutated to phenylalanine residues. This protein was designated as FGFR1-1p since only one tyrosine, Y766, can be phosphorylated in this construct.

All constructs for cloning were PCR amplified using KOD Hot Start polymerase (Novagen) following manufacturer's protocols. All PLCγ1 and FGFR1 variants with deletions and point mutations were introduced by site-directed mutagenesis (QuikChange PCR mutagenesis, Stratagene). All clones ORFs were fully sequenced after manipulation to ensure sequence fidelity.

Expression of recombinant proteins – For expression of Specific Array and smaller domain constructs of PLCγ1 from pTriEx4 or pOPINS vector backbones in rich media. Constructs were transformed into *E.coli* strain C41 (DE3). Colonies were inoculated into 500 ml of 2xYT media and grown to an OD600 of 0.4. Cultures were cooled to 25 °C for 2 hours and expression was induced with 100 μM IPTG for approximately 16 hours. Bacteria were pelleted and stored at -20 °C until processed.

For expression of FGFR1 containing constructs from the pTriEx6 or pOPINS vector backbones in rich media. Constructs were transformed into *E.coli* strain C41 (DE3) harbouring a construct expressing λ-phosphatase. Colonies were inoculated into 500 ml of terrific broth and grown to an OD600 of 1.0. Cultures were cooled to 15 °C and expression was induced with 100 μM IPTG for approximately 16 hours. Bacteria were pelleted and stored at -20 °C until processed.

For expression of Specific Array and smaller domain constructs of PLCγ1 from pTriEx4 or pOPINS vector backbones in minimal medium (for ^{15}N or $^{15}N/^{13}C$ labeling). Constructs were transformed into *E.coli* strain C41 (DE3). Colonies were inoculated into 1.5 ml of LB broth containing antibiotic and grown for 4 hours at 37 °C. Minimal media agar plates were prepared containing the appropriate antibiotic with 3 plates necessary for every 500 ml planned culture. Each plate was spread with 100 μl of the LB culture and bacterial lawns allowed to grow overnight at 37 °C. Bacteria were scraped from the plates into 500 ml of minimal media containing a source of ^{15}N-ammonium or ^{15}N-ammonium and ^{13}C-glucose. Cultures were grown in baffled 2 litre flasks for 1 hour at 37 °C and then 2 hours at 25 °C. The cultures were induced with 100 μM IPTG and grown on for approximately 16 hours. Bacteria were pelleted and stored at -20 °C until processed.

For expression of PLCγ1 core and full-length variants, Freestyle 293F cells were the eukaryotic system of choice. Cells were grown in suspension on a platform shaker in a humidified 37°C CO_2 incubator (Infors) with rotation at 130 rpm. Cells were maintained between 4 x 10^5 and 3 x 10^6 cells/ml in a volume of 250 ml in 1 L culture flasks using Freestyle 293F Expression Medium (Invitrogen). For transfections, 250 ml of 293F cells (1.0 x 10^6 cells) were mixed with plasmid DNA:PEI complexes prepared as follows. 10 ml of OptiPRO SFM™ (Invitrogen) supplemented with 4mM of L-Glutamine was mixed with 312 μg of DNA and a volume of PEI (~25 kDa branched) at 1 mg/ml that is 1.5 times the mass ratio of the amount of DNA. The transfection mix was incubated at room temperature for 15 minutes before being added to the 293F cells. Following incubation for 72 h at 37 °C with shaking, the cells

were pelleted by centrifugation at 2000 x *g* for 15 min. The pellets were snap frozen in liquid nitrogen and then stored at -80 °C.

Purification of recombinant proteins – For purification of protein domains expressed in C41 (DE3). Pellets derived from 1 L cultures were resuspended in 20 ml of chilled Lysis Buffer (25 mM Tris.Cl, 250 mM NaCl, 40 mM Imidazole, 10 mM Benzamidine, 1 mM $MgCl_2$ and 100 μM $CaCl_2$, 100 μg/ml lysozyme, pH 8.0). Resuspension was accomplished by placing the pellets on an orbital shaker set at 200 rpm at 4 °C for 30 minutes. Lysis was continued by the addition of 5 ml of a solution of 10% (v/v) Triton-X-100 and 1 Kunit of bovine pancreatic DNAse I, on the orbital shaker at 200 r.p.m. at 4 °C for 1 hour. Clarification of the lysate was performed by centrifugation of the sample for 1 hour at 4 °C at 18,000 rpm in an SS34 rotor (Sorvall). The clarified sample was applied to a 5 ml HisTrap column (GE Healthcare) on an Akta Explorer system (GE Healthcare) utilizing His Buffer A (25 mM Tris.Cl, 500 mM NaCl, 40 mM Imidazole, 1 mM TCEP, pH 8.0). Non-specifically bound proteins were removed by washing the column with 10 column volumes of His Buffer A. His-tagged recombinant proteins were eluted with a linear gradient from His Buffer A to His Buffer B (25 mM Tris.Cl, 500 mM NaCl, 500 mM Imidazole, 1 mM TCEP, pH 8.0) over 5 column volumes. Eluted protein was quantified using a Nanodrop (Thermo Scientific) using the extinction coefficient of the protein. The N-terminal tags on the proteins were cleaved overnight by addition of the relevant protease, 10 μg of protease was added per 1 mg of His-tagged protein. For pTriEx4-expressed proteins, TeV protease was utilized. For His-Sumo tagged proteins, Ulp1 protease was added. For pTriEx6-expressed proteins the tags were left intact and the subsequent purification steps are outlined in the next paragraph below.

The protein/protease mix was dialysed overnight at 4 °C against 500 ml of Dialysis Buffer (25 mM Tris.Cl, 150 mM NaCl, 10 mM Imidazole and 1 mM TCEP, pH 8.0). Subsequently, proteins were passed again over the 5 ml HisTrap column and material that did not bind was collected. These proteins were dialysed against Low Salt Dialysis Buffer (25 mM Tris.Cl, 20 mM NaCl, 1 mM TCEP, pH 8.0) for a minimum of 3 hours at 4 °C. Subsequently, proteins were further purified by application to a 5 ml HiTrap Q column (GE Healthcare) in Q Buffer A (25 mM Tris.Cl, 20 mM NaCl, 1 mM TCEP, pH 8.0) and eluted in a linear gradient to 50% of Q Buffer B (25 mM Tris.Cl, 1 M NaCl, 1 mM TCEP, pH 8.0) over 25 column volumes. Fractions containing the recombinant protein were pooled and then applied to a Superdex 75 26/60 column (GE Healthcare). The Gel Filtration buffer used depended on the final application for the protein. For NMR analyses the following buffer was used, 25 mM Na_2HPO_4/NaH_2PO_4, 50 mM NaCl, 5 mM DTT, 1 mM EDTA, pH 6.5. For other analyses the standard Gel Filtration Buffer was 25 mM Tris.Cl, 150 mM NaCl, 1 mM TCEP, pH 8.0. Fractions were collected, pooled and concentrated in Vivascience spin concentrators (Vivaproducts), snap frozen in liquid nitrogen and stored at -80 °C.

FGFR1 with 2xStrpII tags as expressed from pTriEx6 were purified differently after the HisTrap purification step. Fractions of these proteins were immediately applied to a 5 ml Streptactin column (GE Healthcare) and washed with Streptactin Buffer A (25 mM Tris.Cl, 150 mM NaCl, pH 8.0) to remove non-specifically bound contaminants. The recombinant proteins were eluted from the column using Streptactin Buffer B (25 mM Tris.Cl, 150 mM NaCl, 5 mM desthiobiotin, pH 8.0). Eluted protein was further purified as above using standard Gel Filtration Buffer, concentrating and storing the protein.

Proteins expressed in 293F cells were isolated by the following method. Cell pellets corresponding to 15 to 25 g of material were resuspended in 25 ml of 293F Lysis Buffer (25 mM Tris.Cl, 250 mM NaCl, 40 mM Imidazole, 10 mM Benzamidine, 1 EDTA free protease inhibitor tablet (Roche), pH 8.0). Cell lysis was performed using a sonicator probe supplying 5 x 30 sec pulses with 30 sec between each pulse while material was kept on ice. Lysed material was clarified by centrifugation at 4 °C for 2 hours at 18,000 rpm in a Sorvall SS34 rotor. Subsequently, proteins were purified as outlined above with the following exceptions. Full-length PLCγ1 proteins were left with their tags intact and after HisTrap purification were purified by Gel Filtration on a Superdex 200 26/60 into standard buffer before concentrating and storing. PLCγ1-core proteins had their tags removed by incubation with HRV 3C protease and were subsequently treated as outlined above except that Superdex 200 26/60 Gel Filtration was applied.

Phosphorylation of FGFR1 for ITC experiments- The human FGFR1 (456-774, L457V, Y463F, C488A, Y583F, C584S, Y585F, Y653F and Y654F) kinase domain that had been expressed as a His-SUMO tagged protein was purified as outlined above. This protein contains only one remaining tyrosine that can be autophosphorylated, Y766, but since the protein was expressed in cells harbouring λ-phosphatase, this protein is largely unphosphorylated. To enable autophosphorylation, the protein was concentrated to 20 mg/ml in 25 mM Tris.Cl, 150 mM NaCl, 1 mM TCEP, pH 8.0. To this protein, 25 mM $MgCl_2$ and 10 mM ATP was added and incubated for 72 hours at 4 °C. The resulting protein was desalted into 25 mM Tris.Cl, 20 mM NaCl, 1 mM TCEP, pH 8.0 and applied to a Resource Q (GE Healthcare)

column. The unphosphorylated and phosphorylated forms were separated by application of a shallow NaCl gradient from 20 mM to 250 mM over 40 column volumes. Non-phosphorylated protein was recycled through the same procedure. Phosphorylated FGFR1^{pY766} designated FGFR1-1p was concentrated, snap frozen in liquid nitrogen and stored at -80 °C.

Phosphorylation of PLCγ1nSH2-cSH2 for ITC experiments and Crystallography- The tandem PLCγ1nSH2-cSH2 (545-790) domains contain 3 tyrosine residues, Y771, 775 and 783, that are readily phosphorylated by FGFR1 and other tyrosine kinases. It was found that phosphorylation and subsequent isolation of preparations phosphorylated at all 3 residues or specifically at one or more residues was practically very challenging. Furthermore, tandem proteins phosphorylated at Y771 or Y775 often form complexes with the SH2 domains form other tandem molecules, leading to aggregation. Therefore, to simplify the procedure and increase the homogeneity of the preparation, 2 of the tyrosine residues, Y771 and Y775 (shown not to be important for activation of PLCγ1), were mutated to phenylalanine. This mutant tandem, nSH2-cSH2^{Y771F,Y775F} was shown to behave identically to the wild-type in pull-down assays. Nevertheless, a method could still not be designed to allow separation of the non-phosphorylated from the pY783-phosphorylated tandem. Instead the following method was applied to maximize the amount of pY783 tandem produced, which relies on the discovery that pY783 tandem (as well as phosphorylated γ1SA and full-length PLCγ1) has a lower affinity for FGFR1 than the non-phosphorylated variants.

Five hundred μl of Streptactin Macroprep beads (IBA) were washed 3 times in Pull-Down Buffer (25 mM Tris.Cl, 150 mM NaCl, 1 mM TCEP, pH 8.0) and subsequently

added to 1.1 mg of FGFR1-3p. After 5 minutes the beads were washed a further 3 times to remove unbound protein. The FGFR1 was activated through the addition of ATP buffer (25 mM Tris.Cl, 150 mM NaCl, 25 mM $MgCl_2$, 10 mM ATP, 1 mM TCEP, pH 8.0) and incubated for 10 minutes at room temperature. Twenty-five mg of PLCγ1nSH2-cSH2^{Y771F,Y775F} was added and phosphorylation continued for 72 hours at 4 °C. Protein in the supernatant was separated from the beads by centrifugation and desalted on a Superdex 75 26/60 column into standard Gel Filtration Buffer. Protein was concentrated and used immediately for setting up crystals or snap frozen in liquid nitrogen and stored at -80 °C in preparation for ITC measurements.

Phosphorylation of PLCγ1 full length (Y186F, Y472F, Y481F, Y771F, Y775F, Y959F, Y977F, Y1254F) for SAXS measurements- Although the wt variant of full length PLCγ1 could be phosphorylated *in vitro* with FGFR1, the resulting protein was prone to aggregation at high concentrations and could not be used in SAXS measurements. To overcome this hurdle, we mutated all the tyrosine phosphorylation sites except Y783 to phenylalanine. The resulting mutant protein was phosphorylated in a manner identical to that outlined above for the tandem mutant except that 15 mg of PLC was used.

Crystallography, NMR and SAXS measurements

Crystallography- Crystals of both apo and phosphorylated (nSH2-cSH2) were prepared by the hanging drop vapor diffusion method. The apo form of the protein was crystallized using 22% PEG 5000 MME, 0.1M Na Malonate and 20mM $CaCl_2$ as a precipitant and the phosphorylated form crystallized in 18 % PEG 8000, 0.1 M HEPES.Na and 200 mM Calcium Acetate, pH 7.5. The protein concentration used

was 10 mg/ml for both crystal forms and plates were incubated at 16°C. The crystals were cryoprotected using 30% ethylene glycol made up in the precipitant solution then added in an equal volume to drops followed by transfer of crystals into the cryosolution prior to flash freezing. Data were collected for the apo form at ID29 at the ESRF and for the phosphorylated form at IO4 at the DIAMOND Synchrotron. The data was processed using the XDS package for integration of the images, the CCP4 package for further processing and the BUSTER package for refinement. Building was carried out with the program COOT. Final images were created using the program PYMOL.

Nuclear Magnetic Resonance- NMR experiments of labeled protein constructs were typically executed in a protein construct concentration range of 0.2-0.5 mM. The titration experiments with unlabeled partner (0-3.0 molar equivalents) were conducted at constant concentration of the labeled component (McAlister et al, 1996). Typical acquisition times for 2D WATERGATE-flipback ^{15}N,^{1}H-HSQC (Grzesiek & Bax, 1993) datasets were ~1 h.

^{15}N Relaxation Measurements- Spin relaxation data were collected at 25° C for a 200 mM ^{15}N-labeled tandem nSH2-cSH2 construct (residues 545-790) on a Bruker AVANCE spectrometer operating at 600MHz. R1 and R2 were collected as previously described (Kay et al, 1989). R1 and R2 values were determined for each residue by fitting an exponential decay to the peak intensity of data collected in an interleaved manner to minimize time dependent temperature or stability effects with delay times in random sequence. T1 longitudinal recovery delays were set to 10, 100, 200, 300, 500, 800, 1000 and 1500 ms. T2 transverse recovery delays were set to 8,

24, 48, 72, 96, 128, 160 and 208 ms. In each case the error was determined from the fit according to a procedure implemented in CCPN Analysis (Vranken et al, 2005). Residues were excluded in which overlap in the data precluded accurate measurement of peak intensity. Isotropic correlation times were determined using the programe TENSOR2 (Dosset et al, 2000). Data for residues where R2/R1 deviated significantly from the bulk, indicative of local motion or chemical exchange, were also excluded from the fits.

Differential line broadening analysis- The trajectory of the cross-peak intensities (inversely related to the contributing linewidths) in multidimensional heteronuclear NMR spectra employed to monitor interactions between macromolecules can reflect contributions from fast, intermediate and slow exchange. For a given cross peak the outcome will depend upon the 'local' magnitudes of the chemical shift difference(s) between the species in solution (including any intermediates along the reaction pathway) and the relevant microscopic rate constants. Wagner and co-workers (Matsuo et al, 1999) recognised that it is useful to profile the effect upon cross-peak linewidths and intensities by measurement of the quantity

$$\Delta_i = (h_{0,i}/\mathbf{H}_0) - (h_i/\mathbf{H}_i)$$

where $h_{0,i}$ and h_i represent the individual cross peak heights from the labeled protein spectrum in the absence and presence of a specific concentration of the binding partner, and $\mathbf{H}_0$ and $\mathbf{H}_i$ represent the average peak heights in the corresponding 2D NMR spectrum (Walters et al, 1999). A non-uniform profile of the quantity Δ_i (Figure 5B in the main manuscript) across the polypeptide chain would indicate differential

line broadening in the binding reaction potentially highlighting regions of the labelled protein that are most strongly perturbed in the encounter with the binding partner.

SAXS data collection and analysis- Synchrotron SAXS data were collected on the EMBL X33 camera with a Pilatus detector on the storage ring DORIS III (Deutsches Elektronen-Synchrotron (DESY), Hamburg, Germany) and ESRF ID14-3 camera with a Pilatus detector (ESRF, Grenoble, France). All samples were measured for at least three solute concentrations ranging from 1 mg/ml to 1-10 mg/ml. The data were processed by the program PRIMUS (Konarev et al, 2003) following standard procedures (Svergun & Koch, 2002) to compute the radii of gyration (Rg) and maximum dimensions (Dmax). The distance distribution functions, p(r), were evaluated using the program GNOM (Svergun, 1992). The molecular masses of the solutes were estimated by calibration against reference solutions of bovine serum albumin. The excluded particle volumes Vp were computed from the scattering data using Porod invariant (Porod, 1982).

Low resolution models of PLCγ fragments were generated by the *ab initio* program DAMMIN (Svergun, 1999), which represents a protein by volume filled with the packed spheres referred to as dummy atoms. The results of 10 independent DAMMIN runs were analyzed and averaged by SUPCOMB (Kozin & Svergun, 2001) and DAMAVER (Volkov & Svergun, 2003).

Multiple fitting and pairwise comparison of SAXS data for different constructs have been determined by *ab initio* program MONSA (Svergun, 1999) which is an extended version of DAMMIN for multiphase bead modeling and allows to fit simultaneously multiple curves. MONSA reads in multiple data sets and information about the presence or absence of components in the construct.

XPLOR-NIH Protocol to generate random structures for EOM- The SH3 (residues 791-852, PDB 1HSQ), splitPH (residues 488-521 and 874-933, PDB 2FLJ) and tandem nSH2-cSH2 domains (residues 549-767, crystal structure) were treated as rigid bodies. The tandem SH2 structure was held fixed in space whilst the linker regions were given Cartesian degree of freedom. The generation of the structures comprised 4 steps.

Step 1. The relative positions and rotations of the globular domains were randomized, breaking the bonds between the linking segments and the domains (residue pairs 521/522, 548/549, 767/768, 790/791, 852/853, 873/874). Backbone torsion angles in the linker regions were also randomized.

Step 2. The highly distorted bonds linking the randomized linker regions and the globular domains were reconstructed . The geometry of the linker regions was then optimized, allowing the domains to rotate and translate as rigid bodies and with full Cartesian degree of freedom for the linker regions, by 3 cycles of minimization (1000 steps each) to optimize the bond, angles and improper terms.

Step 3. A final gradient minimization was performed with potential energy terms that include bonds, angles, improper torsions, van der Waals and Ramachandran database potential terms allowing the splitPH and SH3 domains to move relative to the tandem nSH2-cSH2 as rigid bodies.

Step 4. Simulated annealing with temperature from 3000 to 25 K is performed with steps of 25K. The dynamics runs for 800 ps or 8000 variable time steps (whichever ends first) and includes all the potentials.

For the restrained model pool, highly ambiguous inter-residue distance restraints were created corresponding to the perturbed resonances in the splitPH and cSH2 domains according to a procedure described by Clore *et al.* (Clore & Schwieters, 2003). The distance restraints were applied using a soft asymptotic square-well energy function with restraints distances calculated as sum-averages over all relevant atom selection pairs, following the randomization of the relative domains positions and prior to reforming the peptide bonds with the linker segments. Full Cartesian degree of freedom was given to the side chains of the residues undergoing chemical shift perturbation.

For clarity, the XPLOR-NIH SAXS and ratio of gyration potential energy terms were not used in the generation of the unrestrained and restrained EOM model pools.

XPLOR-NIH Protocol to generate docked starting structure for ensemble refinement- In addition to the EOM approach we separately attempted to exploit the ensemble modelling approaches to fitting SAXS data provided within the advanced features of the XPLOR-NIH v2.27 software package. Thus instead of using a genetic algorithm to filter a pre-generated pool of conformations for sets that together provide a good fit to the SAXS data, the dynamical simulated annealing target function of XPLOR-NIH can be made to include the experimental SAXS scattering data as part of the target function (along with other experimental restraints) whilst simultaneously requiring the target function to be fit for an ensemble of multiple structural models.

The procedure to generate the starting structures for the XPLOR-NIH ensemble refinement was very similar to the approach adopted for the early stage of the EOM approach (see above). Thus, starting from a hand-built model of the SA structure, the bonds between the globular domains and linking segments were broken, the linker

backbone torsion angles randomized, and the bonds reformed under the influence of the SAXS and NOE (see below) potential with a procedure similar to that adopted by Schwieters *et al*. (Schwieters et al, 2010).

A simulated annealing procedure including all the potentials (including that for backbone torsion angles) was performed similarly to the generation of the random SA structures for EOM.

The docking procedure was repeated five times and the output structures were scored on the basis of their SAXS and NOE energies. Of these the best model was selected for further calculations. Overall a total of 200 conformers were generated for analysis and subsequent ensemble refinement.

Similarly to the EOM method we model transient interactions between the cSH2 and spPH domains using highly ambiguous NOE restraints involving all the backbone and side chain atoms of the residues that display chemical shift perturbations in the NMR spectra (as above).

XPLOR-NIH Ensemble refinement- Of the 200 starting conformers generated from the docking procedure the 50 conformers with the lowest NOE RMSD (< 2.25 Å) were selected for ensemble refinement against the SAXS data.

To select the smallest number of ensemble members to reproduce the experimental data, refinement of a subset of structure was done with ensemble number (Ne) of 2, 4, 8 and 16. There was no improvement in the calculated NOE and SAXS RMSDs using Ne > 4, therefore for the refinement stage Ne=4 was used. The 50 selected conformers were subjected to a simulated annealing procedure with temperature ramped down from 3000 to 25 K in 25 K steps. The included potential terms were averaged over the ensemble. (For clarity, the Radius of gryration (Rg) and ‘restrain atom positions’

(RAP) potential terms were not used. The use of Rg potential would constrain each member of the ensemble to have the same radius of gyration whilst the RAP potential is used to keep the ensemble members from drifting too far apart.) Full torsional freedom was given to the linkers (that reduces the convergence but increases the probability that the ensemble members will have different domain arrangements).

At the end of the calculation the 25 four-member ensembles that best agree with the experimental SAXS data were selected for structural analysis (a total of 100 structural models).

As the number of calculated conformations is large, a weighted atomic probability density map was used to represent the relative domain positions. A volumetric mass density map was calculated corresponding to the atoms of the spPH domain using the program VMD (Humphrey et al, 1996). The map represents the variation of the position of the spPH domain relative to that of the tandem nSH2-cSH2 domains (which was held fixed during the calculations). The volumetric map was calculated for the residues 488-521 and 874-933 over a 3Å resolution grid with an effective atomic size taken corresponding to three times the corresponding standard atomic radius. The volumetric map was weighted according to the atomic masses to obtain the overall mass distribution and represented as a wireframe isosurface.

Functional Assays

Analysis of inositol phosphate formation in intact COS-7 and PAE cells- COS-7 and PAE cells were maintained at 37 ^{o}C in a humidified atmosphere of 95 % air and 5 % CO_2 in either Dulbecco's modified Eagle's medium (DMEM) (Invitrogen) for COS-7 or Ham's F12 (Invitrogen) for PAE supplemented with 10 % (v/v) foetal bovine serum (Invitrogen) and 2.5 mM glutamine. Prior to transfection, cells were seeded

into 6-well plates at a density of 1.7x10^5 cells/well (COS-7) or 3x10^5 cells/well (PAE) and grown for 24 h in 2 ml/well of the same medium. For transfections, 1.0 μg (COS-7) or 1.5 μg (PAE) of PLCγ DNA was mixed with 1 μl PlusReagent™ and 7 μl Lipofectamine™ (Invitrogen) and added to the cells in 0.8 ml media without serum. The cells were incubated for 3 hours (COS-7) or 2 hours (PAE) at 37 °C, 5% CO_2 before the transfection mixture was removed and replaced with media containing serum. Twenty-four hours (COS-7) or 6 hours (PAE) post-transfection cells were labeled with 1.5μ Ci/ml *myo*- [2-^{3}H] inositol (Perkin-Elmer). After a further 24 h the cells were incubated in 1.2 ml inositol free media without serum containing 20 mM LiCl with or without stimulation with EGF (COS-7) or FGF/Heparin (PAE) for 1 h. The cells were lysed by addition of 1.2 ml 4.5 % perchloric acid and supernatants and pellets were separated. Inositol phosphates were collected using AG1-X8 200–400 columns (BioRad). Levels of inositol phosphates were quantified by liquid scintillation counting using Ultima-Flo scintillation fluid (Perkin Elmer). PLC activity is expressed as the total inositol phosphates formed relative to the amount of [^{3}H] inositol in the phospholipid pool.

Isothermal Titration Calorimetry- For FGFR1-1p interactions with PLCγ1 domains. Heats of interaction were measured on a VP-ITC system (Microcal) with a cell volume of 1.458 ml. Proteins were dialysed for 16 hours in ITC buffer (25 mM Tris.Cl, 150 mM NaCl, 1 mM TCEP, pH 8.0). PLCγ1 domains were loaded in the sample cell at 25 μM and titrated with FGFR1-1p in the syringe (400 μM). The titrations were performed while samples were being stirred at 260 r.p.m. at 20 °C. A total of 20 injections was carried out with 15 μl injected each time (except the first injection when 3 μl was injected) and a 4 min interval between each injection to allow

the baseline to stabilise. The data were fitted with either a two-site or single site (where appropriate) model to calculate the number of binding sites (n), the binding constant (K_a), the change in enthalpy (ΔH^o) and change in entropy (ΔS) using Origin software (Microcal).

For PLCγ1-core interactions with the cSH2 domain. Heats of interaction were measured on an iTC_{200} system (Microcal) with a cell volume of ~200 μl. Proteins were dialysed for 16 hours in ITC buffer (25 mM Tris.Cl, 150 mM NaCl, 1 mM TCEP, pH 7.5). Proteins were loaded in the sample cell at ~100 μM and titrated with the binding partner in the syringe (~1 mM). The titrations were performed while samples were being stirred at 1000 r.p.m. at 20°C. A total of 20 injections was carried out with 2 ml injected each time (except the first injection when 0.4 μl was injected) and a 1 min interval between each injection to allow the baseline to stabilise. The data were fitted with a single site model to calculate the number of binding sites (n), the binding constant (K_a), the change in enthalpy (DH^o) and change in entropy (DS) using Origin software (Microcal).

Pull Down experiments- The pull down assay was used to immobilize FGFR1-3p proteins and pull down PLCγ1 constructs (full length or γSA). To bind 2xStrpII-tagged FGFR1-3p to Streptactin Macroprep beads (IBA), 57 μg of FGFR1-3p was immobilized on 300 μl of Streptactin Macroprep beads in Pull Down Buffer (PDB) (50 mM Tris.Cl, 150 mM NaCl, 25 mM $MgCl_2$, 1 mM $MnCl_2$, 2 mM TCEP, 0.1 % (v/v) Triton-X-100, pH 8.0) to which 10 mM ATP had been added. Incubation was carried out at room temperature for 1 hour with constant agitation. The beads were subsequently washed five times in 500 μl of PDB to remove ATP and any unbound

protein and then resuspended in 1.1 ml of PDB. The PLCγ1 molecules were diluted to 0.3 mg/ml (Full length) or 0.6 mg/ml (γSA). Subsequently, to each microfuge tube the following were added, 100 μl of FGFR1-3p loaded beads, 500 μl of PDB containing either 10 mM ATP or not, 50 μl of the PLCγ1 construct and incubated at room temperature for 1 hour with constant agitation. The beads were then washed five times in PDB and then were boiled in 50 μl Laemmli buffer and a 20 μl aliquot subjected to SDS-PAGE. Gels were stained with Colloidal Coomassie and imaged with a Syngene G-Box gel documentation system. Bands were quantified using ImageJ software.

In-vitro PLC assay- Experiments to determine the effect of phosphorylation or the removal of the regulatory γSA on PLC activity used phospholipid vesicles containing 33 mM PtdIns(4,5)P_2, 536 μM PE, and ~15,000 cpm/assay [^{3}H]PtdIns(4,5)P_2 (Perkin Elmer). Lipid were dried down under a stream of N_2 and then resuspended in reaction buffer. Lipid vesicles were formed by a combination of vortexing and cup sonication. Final buffer conditions were 50 mM Tris.Maleate (pH 7.3), 70 mM KCl, 3 mM EGTA, 2 mM DTT, 0.18 % (w/v) fatty acid free-BSA, and 300 nM free Ca^{2+} in a final volume of 60 μl. All reactions were performed at 30°C for 45 min. Reactions were terminated through the addition of 350 μl of Stop solution I [Chloroform:Methanol:HCl (500:500:3)] and 100 μl of Stop Solution II (1M HCl, 5mM EGTA) and [^{3}H]Ins(1,4,5)P_3 was quantified by liquid scintillation counting of the soluble fraction after centrifugation of the reaction mixture.

Mass Spectrometry analysis of peptides- Proteins were digested directly in excised fragments from SDS-PAGE gels using trypsin or other proteases. Samples were

infused into an LTQ Velos Orbitrap mass spectrometer (Thermo Fisher Scientific, Hemel Hempstead, UK). MS/MS were acquired using data dependent acquisition to sequence the top 10 most intense ions using enhanced ion trap scans. Raw MS/MS data was submitted for database searching using Proteome Discoverer v1.2 and Mascot v2.2. MS/MS-based peptide and protein identifications were grouped and validated using Scaffold v3.0 (Proteome Software Inc., Portland, OR). For characterization of phosphorylation sites the spectra visually inspected to evaluate the accuracy in localisation of the assigned modification(s).

SUPPLEMENTAL REFERENCES

Clore, G.M., and Schwieters, C.D. (2003). Docking of protein-protein complexes on the basis of highly ambiguous intermolecular distance restraints derived from 1H/15N chemical shift mapping and backbone 15N-1H residual dipolar couplings using conjoined rigid body/torsion angle dynamics. J Am Chem Soc *125*, 2902-2912.

Dosset, P., Hus, J.C., Blackledge, M., and Marion, D. (2000). Efficient analysis of macromolecular rotational diffusion from heteronuclear relaxation data. Journal of biomolecular NMR *16*, 23-28.

Grzesiek, S., and Bax, A. (1993). Amino acid type determination in the sequential assignment procedure of uniformly 13C/15N-enriched proteins. Journal of biomolecular NMR *3*, 185-204.

Humphrey, W., Dalke, A., and Schulten, K. (1996). VMD: visual molecular dynamics. Journal of molecular graphics *14*, 33-38, 27-38.

Kay, L.E., Torchia, D.A., and Bax, A. (1989). Backbone dynamics of proteins as studied by 15N inverse detected heteronuclear NMR spectroscopy: application to staphylococcal nuclease. Biochemistry *28*, 8972-8979.

Konarev, P.V., Volkov, V.V., Sokolova, A.V., Koch, M.H.J., and Svergun, D.I. (2003). PRIMUS: a Windows PC-based system for small-angle scattering data analysis. J Appl Crystallogr *36*, 1277-1282.

Kozin, M.B., and Svergun, D.I. (2001). Automated matching of high- and low-resolution structural models. J Appl Crystallogr *34*, 33-41.

Matsuo, H., Walters, K.J., Teruya, K., Tanaka, T., Gassner, G.T., Lippard, S.J., Kyogoku, Y., and Wagner, G. (1999). Identification by NMR spectroscopy of residues at contact surfaces in large, slowly exchanging macromolecular complexes. J Am Chem Soc *121*, 9903-9904.

McAlister, M.S., Mott, H.R., van der Merwe, P.A., Campbell, I.D., Davis, S.J., and Driscoll, P.C. (1996). NMR analysis of interacting soluble forms of the cell-cell recognition molecules CD2 and CD48. Biochemistry *35*, 5982-5991.

Porod, G. (1982). Citation Classic - X-Ray Low-Angle Scattering of Dense Colloid Systems .1. Cc/Phys Chem Earth, 22-22.

Schwieters, C.D., Suh, J.Y., Grishaev, A., Ghirlando, R., Takayama, Y., and Clore, G.M. (2010). Solution structure of the 128 kDa enzyme I dimer from Escherichia coli and its 146 kDa complex with HPr using residual dipolar couplings and small- and wide-angle X-ray scattering. J Am Chem Soc *132*, 13026-13045.

Svergun, D.I. (1992). Determination of the Regularization Parameter in Indirect-Transform Methods Using Perceptual Criteria. J Appl Crystallogr *25*, 495-503.

Svergun, D.I. (1999). Restoring low resolution structure of biological macromolecules from solution scattering using simulated annealing. Biophys J *76*, 2879-2886.

Svergun, D.I., and Koch, M.H. (2002). Advances in structure analysis using small-angle scattering in solution. Current opinion in structural biology *12*, 654-660.

Volkov, V.V., and Svergun, D.I. (2003). Uniqueness of ab initio shape determination in small-angle scattering. J Appl Crystallogr *36*, 860-864.

Vranken, W.F., Boucher, W., Stevens, T.J., Fogh, R.H., Pajon, A., Llinas, M., Ulrich, E.L., Markley, J.L., Ionides, J., and Laue, E.D. (2005). The CCPN data model for NMR spectroscopy: development of a software pipeline. Proteins *59*, 687-696.

Walters, K.J., Gassner, G.T., Lippard, S.J., and Wagner, G. (1999). Structure of the soluble methane monooxygenase regulatory protein B. Proceedings of the National Academy of Sciences of the United States of America *96*, 7877-7882.
